# Cryotherapy in joint arthroplasty rehabilitation: Effects on pain, analgesic consumption, blood loss, and range of motion

**DOI:** 10.1097/MD.0000000000046802

**Published:** 2026-01-02

**Authors:** Yu Xie, Lijun Wang, Shizheng Du, Weiyu Pan, Junjuan Zhang, Xin Li

**Affiliations:** a Department of Orthopedics, Henan Provincial Intelligent Nursing and Transformation Engineering Research Center, Henan Provincial Key Medicine Laboratory of Nursing, Henan Provincial People’s Hospital, Zhengzhou University People’s Hospital, JBI Henan Evidence-based Nursing Centre, University of Adelaide, Zhengzhou, Henan, China; b Department of Rehabilitation, Henan Provincial Intelligent Nursing and Transformation Engineering Research Center, Henan Provincial Key Medicine Laboratory of Nursing, Henan Provincial People’s Hospital, Zhengzhou University People’s Hospital, JBI Henan Evidence-based Nursing Centre, University of Adelaide, Zhengzhou, Henan, China; c School of Nursing, Nanjing University of Chinese Medicine, Nanjing, Jiangsu, China.

**Keywords:** cryotherapy, meta-analysis, randomized controlled trial, systematic review, total joint arthroplasty

## Abstract

**Background:**

Effectiveness of cryotherapy in the rehabilitation of joint arthroplasty remains controversial. Our systematic review was to evaluate application of cryotherapy following total joint arthroplasty on outcome measures related to rehabilitation exercise.

**Methods:**

PubMed, Web of Science, Cochrane Library, Embase, and CIHAHL, Grey literature, and other relevant databases for randomized controlled trials were searched. A meta-analysis was performed using Revman5.3, and we used Cochrane risk-of-bias 2.0 (RoB2.0) tool to evaluated the quality of included literature. We also use GRADEprofiler 3.6 software for evidence quality assessment.

**Results:**

A systematic review of 21 studies, with a pooled analysis of 1462 patients (1177 total knee arthroplasty [TKA] and 285 total hip arthroplasty [THA]), was conducted. All assessed studies were deemed eligible for inclusion. Compared to non-cryotherapy group, meta-analysis revealed that cryotherapy following TKA significantly alleviated postoperative pain on the 2nd day and reduced analgesic consumption (*P*<.05). Additionally, cryotherapy was associated with a mitigation of hemoglobin changes, a reduction in blood loss infiltrating subcutaneous soft tissue, and a decrease in drainage tube output (*P*<.05), collectively contributing to a reduction in postoperative blood loss (*P*<.05). In the subgroup analysis focusing on THA, cryotherapy was found to significantly reduce pain perception on postoperative day 1 and day 2 (*P*<.05). However, no significant effects were observed on analgesic consumption or blood loss reduction (*P*>.05). Notably, cryotherapy did not enhance range of motion as initially hypothesized. Subgroup analyses revealed that cryotherapy did not improve range of motion at 1 week post-TKA, on day 1 post-THA, or on day 2 post-THA (*P*>.05). Furthermore, cryotherapy did not increase the risk of adverse events (*P*>.05). Additionally, cryotherapy showed no significant impact on enhancing patient satisfaction with rehabilitation, reducing joint swelling, decreasing transfusion rates, or shortening hospital stays (*P*>.05). The GRADE assessment determined that the quality of evidence for these outcomes ranged from very low to moderate.

**Conclusion:**

Cryotherapy has the potential to aid in joint rehabilitation following total joint arthroplasty. However, it is essential to consider the specific type of joint replacement surgery the patient has undergone, as the rehabilitative effects of cryotherapy differ between TKA and THA. The variability in the quality of the studies included, along with limited sample sizes and generally low quality evidence, necessitates further validation of these findings through rigorously designed, large-scale, multicenter randomized controlled trials.

## 1. Introduction

Total joint arthroplasty (TJA) is a well-established and definitive surgical treatment for end-stage joint diseases.^[1,2]^ As a broad procedural category, TJA includes arthroplasties of the knee, hip, shoulder, elbow, finger, and ankle. Among these, total knee arthroplasty (TKA) and total hip arthroplasty (THA) are the most commonly performed, with the goals of restoring joint function, relieving pain, and improving quality of life.^[3]^ It is projected that by 2030, the annual number of total hip and knee replacement procedures in the United States will exceed 1.5 million.^[4]^ Nevertheless, the early postoperative course is frequently complicated by surgical pain, inflammatory reactions, hemarthrosis, and periarticular swelling. These complications often lead to greater reliance on analgesics, increased transfusion needs, and reduced joint mobility, which collectively delay functional recovery, extend hospital stays, and raise medical costs.^[1,5,6]^ Although surgical techniques and multimodal analgesia have advanced, TJA remains challenging for many patients.^[7]^ Thus, optimizing postoperative recovery and rehabilitation continues to be a priority for clinical teams involved in TJA care.^[8]^

The rehabilitation process after TJA is profoundly influenced by the quality of postoperative pain control, where suboptimal management can lead to increased opioid use, restricted motion, and delayed mobilization.^[9]^ This has accelerated the adoption of non-pharmacological strategies, including cryotherapy,^[10]^ electrical stimulation,^[9]^ acupuncture,^[11]^ and resistance training.^[12]^ Comparative analyses, however, reveal notable limitations: electrical stimulation is often poorly tolerated at effective intensities,^[8]^ acupuncture’s invasiveness and need for expertise hinder scalability,^[11]^ and resistance training outcomes vary substantially with patient compliance.^[12]^ Consequently, cryotherapy has gained prominence in orthopedic practice attributable to its non-invasiveness, operational simplicity, and low adverse effect incidence.^[13]^ Notwithstanding its widespread use, a rigorous and contemporary synthesis of evidence regarding its impact on core rehabilitation endpoints is essential to consolidate clinical guidance. Cryotherapy, which involves the localized application of cold via ice packs or chilled fluids, is commonly employed following TJA to mitigate surgical tissue trauma. Its therapeutic mechanism is attributed to cold-induced vasoconstriction, which reduces local blood flow, minimizes postoperative bleeding, and attenuates inflammation and edema in the periarticular soft tissues.^[14]^ Furthermore, by lowering intra-articular temperature, cryotherapy may slow nerve conduction velocity and modulate pain perception, thereby contributing to enhanced postoperative comfort. Despite its widespread clinical use and plausible physiological basis, the empirical evidence supporting cryotherapy in TJA remains inconsistent. Previous studies, predominantly focused on TKA, have reported conflicting results regarding its effects on key recovery metrics such as pain levels, analgesic consumption, blood loss, and range of motion (ROM).^[10,15–17]^ Moreover, as the scope of TJA continues to expand to include THA, total shoulder arthroplasty, and total ankle arthroplasty procedures, it is unclear whether the benefits observed in TKA are generalizable across other joint types. In addition, existing outcome measures often lack comprehensiveness, failing to fully capture the multidimensional nature of functional recovery. Importantly, previous systematic reviews have not consistently conducted rigorous quality assessments of the evidence for specific outcomes, limiting the validity and clinical applicability of their conclusions. Therefore, a methodologically rigorous and comprehensive systematic evaluation is warranted to clarify the therapeutic role of cryotherapy in contemporary TJA practice, particularly across diverse surgical sites and with attention to clinically meaningful recovery endpoints.

Therefore, this study will conduct a systematic review to synthesize current evidence on cryotherapy following TJA, focusing on rehabilitation-relevant outcomes such as postoperative pain, analgesic use, ROM, and blood loss. Secondary outcomes (including patient satisfaction, postoperative swelling, transfusion rate, length of hospital stay, and adverse events) will also be analyzed to evaluate the overall efficacy and safety of cryotherapy. The findings are expected to inform evidence-based postoperative rehabilitation guidelines and support clinical decision-making regarding cryotherapy in TJA recovery.

## 2. Methods

### 2.1. Registration

Study strictly followed the preferred Reporting Items for Systematic review and Meta-Analyses (PRISMA) guideline. The research protocol has registered on the International Prospective Register of Systematic Reviews (PROSPERO) (registration number CRD42022356859). This manuscript is a systematic review, and ethical approval is not necessary.

### 2.2. Inclusion and exclusion criteria

Studies could only be included if they satisfied the following PICOS criteria:

(1) P (population): adult patients older than or equal to 18 years, proposed for primary artificial joint arthroplasty, include TKA, THA, total shoulder arthroplasty, total ankle arthroplasty, total elbow arthroplasty, total finger arthroplasty.
  Exclusion criteria were patients who underwent revision TJA, unicompartmental knee arthroplasty, patients with coagulation disorders, patients with neurological disorders, patients taking high doses of anticoagulants, and comorbidities such as cardiovascular disease, preexisting pain, cancer, or hypertension. Patients with cold allergies, intolerances, or skin abnormalities such as hives were also excluded.
(2) I (intervention): intervention in the experimental group constituted any local joint cryotherapy (encompassing devices, ice bags, or gel packs) founded on the same core principle of action, with ice baths expressly excluded. No minimum duration of application or temperature was stipulated.
(3) C (control): the control group include routine nursing measures (i.e., usual care or treatment as usual); no any postoperative interventions; non-cryotherapy measures, such as compressive bandage, continuous passive machine (CPM).
(4) O (outcome): the main outcome measures that are closely related to the rehabilitation of joint motion function in patients undergoing TJA, including postoperative pain, analgesic use, ROM of joints, blood loss. The additional outcomes includes patients satisfaction, postoperative edema, transfusion rate, hospital length of stay, and adverse events occurrence.
(5) S (study design): randomized controlled trial (RCT).

### 2.3. Search strategy

A comprehensive search of electronic databases was conducted, including PubMed, Web of Science, the Cochrane Library, EMBASE, and Cumulative Index of Nursing and Allied Health Literature (CIHAHL), to identify RCTs evaluating cryotherapy in patients undergoing joint arthroplasty. The search covered the period from database inception to September 12, 2022. An updated search from September 13, 2022 to the present date was also performed but identified no additional eligible studies. The search strategy was developed using a combination of Medical Subject Headings and text words, with key terms including “arthroplasty” AND “cryotherapy” AND “randomized controlled trial.” The strategy was refined through preliminary searches and adapted for each database’s specific requirements. To ensure literature saturation, manual reference checking and backward citation tracking were additionally employed. Two investigators independently screened all retrieved records for eligibility. The complete search strategies and results for each database are documented in Appendix 1, Supplemental Digital Content, https://links.lww.com/MD/Q995.

### 2.4. Selection of studies

All identified citations were managed using EndNote X9 (Clarivate Analytics). Two authors (Y.X. and L.J.W.), who have received formal training in systematic review methodology, independently conducted the study selection process. After removing duplicates, they screened titles and abstracts to identify potentially eligible studies. The full texts of these potentially relevant articles were then retrieved and thoroughly assessed for final inclusion.

The exclusion criteria were applied as follows:

(1) duplicate publications or multiple reports based on the same study population;
(2) publications available only as abstracts with insufficient data, or where the full text could not be obtained;
(3) studies from which relevant outcome data could not be extracted or utilized for analysis.

### 2.5. Risk of bias assessment

Two reviewers (YX and LJW) independently evaluated the methodological quality of the included RCTs using the Cochrane Revised Risk of Bias Tool (RoB 2.0).^[18]^ This tool assesses 5 domains, with each trial receiving an overall and domain-specific classification of “low risk,” “some concerns,” or “high risk” of bias.^[19]^ The reviewers performed assessments independently using standardized forms. After completing their evaluations, they cross-checked the results. Any disagreements were resolved through consensus or by consulting a third reviewer (SZD).

### 2.6. Data extraction

Two authors (YX and LJW) independently extracted data from the included studies using a pre-designed data extraction form. The following variables were recorded: first author and publication year; country; study design; joint arthroplasty type; patient age and sex; experimental group intervention and sample size; control group intervention and sample size; and outcome measures. After independent extraction, a verification process was conducted. One reviewer entered all data into an Excel spreadsheet, while the 2nd reviewer cross-checked the entries against the original articles for accuracy. Discrepancies were resolved through discussion, with unresolved issues adjudicated by a third reviewer (SZD).

### 2.7. Statistical analysis

Statistical analyses were performed using Review Manager (RevMan) version 5.3 (The Cochrane Collaboration, London, United Kingdom). Continuous outcomes are presented as mean difference (MD) or standardized mean difference (SMD) with 95% confidence intervals. For postoperative pain outcomes, which were measured using different scales (Visual Analogue Scale and Numerical Rating Scale), SMD was employed to combine results; these scales were considered comparable based on established correlations.^[20,21]^ MD was used for outcomes reported with uniform units, including blood loss, ROM, and hospital length of stay. Other continuous variables (analgesic consumption, knee circumference, patient satisfaction) were analyzed using SMD. Dichotomous outcomes (transfusion rate, adverse events) are expressed as risk ratios with 95% confidence intervals. All analyses were based on postintervention means and SD.

### 2.8. Assessment of heterogeneity

Firstly, heterogeneity test is carried out. If *P* ≥ .1 and *I*^2^ ≤ 50%, we used fixed-effects model to calculate the combined volume; if *P* < .1 and *I*^2^ > 50%, we used random-effects model for meta-analysis.^[13]^ In the forest plot, a pooled effect size with *P* < .05 indicates a statistically significant difference.

### 2.9. Subgroup analysis and sensitivity analysis

Sensitivity analyses were performed using both fixed-effect and random-effects models for outcomes with significant heterogeneity.^[22]^ The results remained consistent across both modeling approaches. Subgroup analyses were conducted to evaluate potential differences according to the type of joint arthroplasty performed. Publication bias was assessed through visual inspection of funnel plots for outcomes incorporating data from more than 10 studies.

### 2.10. Assessment of evidence quality

Two reviewers (YX and LJW) independently assessed the quality of evidence for each outcome according to the GRADE framework.^[23]^ Evaluations were performed using GRADEpro GDT software (version 3.6; Evidence Prime Inc., Hamilton, ON, Canada). While RCTs initially represent high-quality evidence, the certainty of evidence for each outcome was systematically rated against 5 key domains and classified into 1 of 4 levels: high, moderate, low, or very low. Disagreements between reviewers were resolved through discussion. Persistent disagreements were adjudicated by a 3rd reviewer (SZD) with expertise in evidence-based medicine.^[24]^

## 3. Results

### 3.1. Literature search

**Figure 1.**
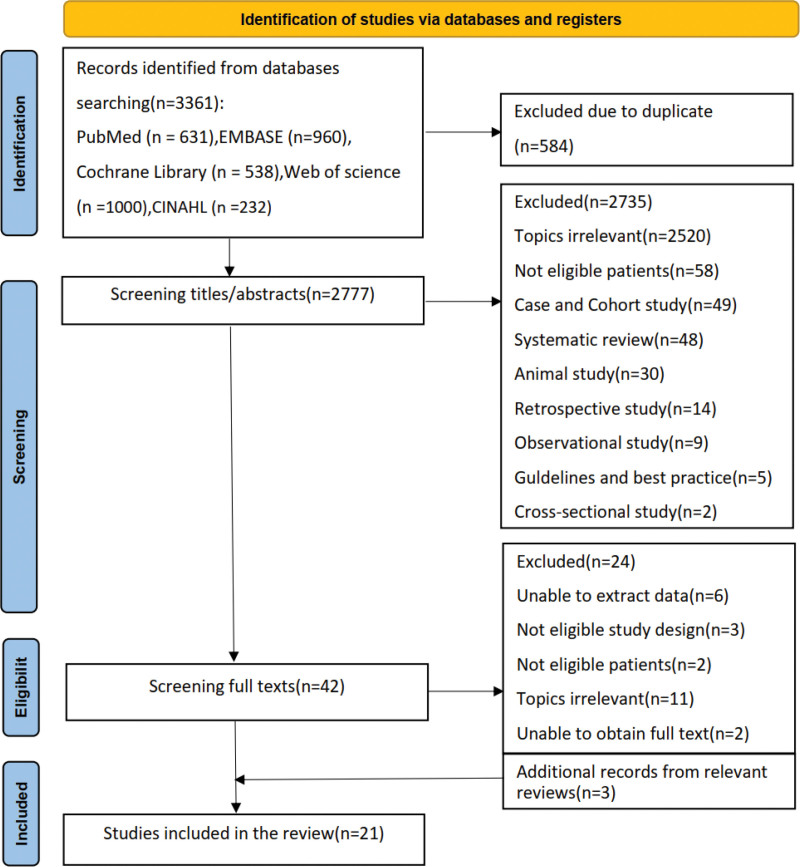
Literature screening process.

The systematic search identified 3361 records from the selected databases. After removal of 584 duplicates through EndNote X9, 2777 unique citations underwent title and abstract screening. This led to the exclusion of 2735 records that did not meet inclusion criteria, primarily due to irrelevant topics, ineligible populations, or non-randomized study designs. Full-text review was conducted for the remaining 42 articles, resulting in the exclusion of 24 studies with specific reasons. Three additional eligible studies were identified through reference tracking. The final analysis included 21 RCTs evaluating cryotherapy following TKA or THA. No studies were found addressing cryotherapy after other joint arthroplasties (ankle, shoulder, elbow, or finger). These included studies, published between 1991 and 2022, encompassed 1177 TKA cases and 285 THA cases. The complete study selection process is summarized in Figure 1.

### 3.2. Study characteristics

**Figure 2.**
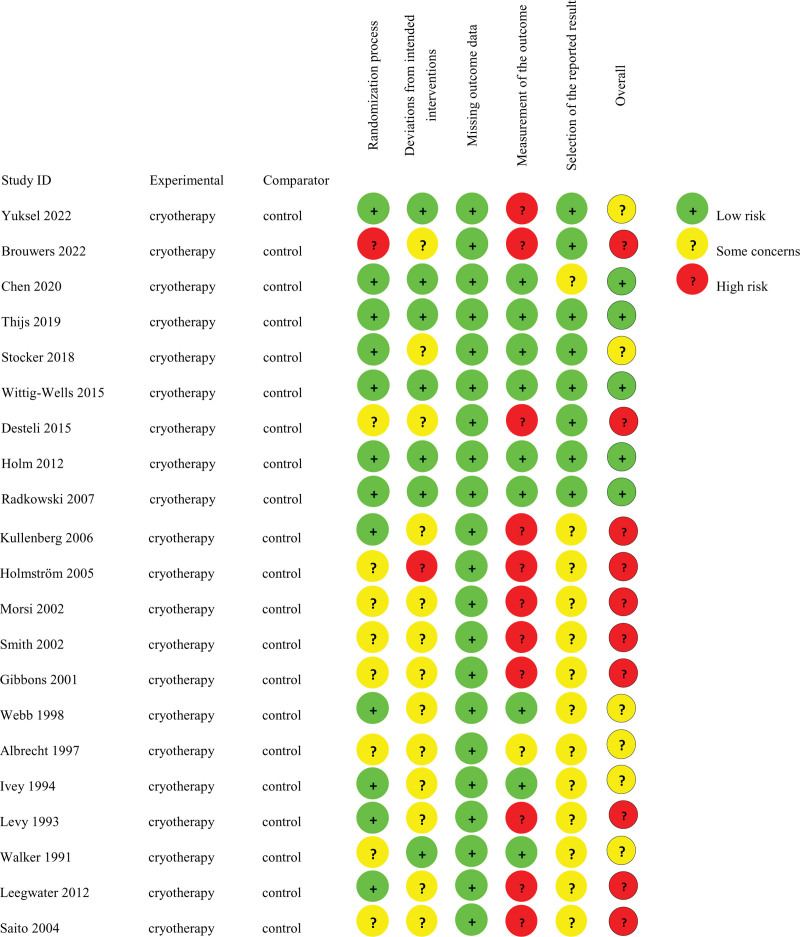
Summary of risk of bias judgements for each study.

**Figure 3.**
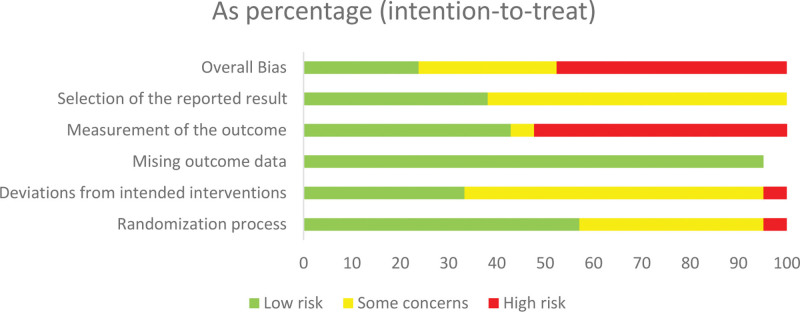
Summary of risk of bias judgements presented as percentages across all included studies.

The 21 included RCTs were conducted in 12 countries: United States, China, Switzerland, Turkey, Germany, Denmark, Sweden, Egypt, Netherlands, Australia, United Kingdom, and Japan. Study characteristics are detailed in Table 1,^[25–45]^ and quality assessment results are presented in Figures 2 and 3. Interventions and controls varied across studies. Sixteen trials compared cryotherapy devices against non-cryotherapy controls, while 5 studies evaluated simple cold packs versus non-cryotherapy.^[26,27,29,30,32]^ Control conditions included no intervention, compression alone, CPM, or combined compression and CPM (Table 1). Given the shared therapeutic mechanism of action across cryotherapy modalities, we pooled data from both device-based and cold pack interventions for comparison against non-cryotherapy groups. While this approach may introduce some heterogeneity, the relatively small proportion of cold pack studies (5/21, 23.8%) suggests this effect is likely minimal.

**Table 1:** Characteristics of included study.

Study	Years	Country	Type of joint arthroplasty	Age		Sample size		Gender (M/F)		Interventions		Outcomes
				CT	C	CT	C	CT	C	CT	C	
Brouwers et al^[18]^	2022	Netherlands	TKA	69.2 ± 6.8	69.6 ± 9.1	49	51	22/27	22/29	Cryotherapy device	Nothing	①②③⑤⑥⑧⑩
Yuksel et al^[19]^	2022	Turkey	TKA	66.8 ± 9.8	65.39 ± 8.94	33	34	10/23	11/23	Cryotherapy(cold packs)	Nothing	①④⑥⑩
Chen et al^[20]^	2020	China	TKA	–	–	30	30	7/23	5/25	Cryotherapy + CPM	Nothing	①③④⑥
Thijs et al^[21]^	2019	Netherlands	TKA	65.5 ± 6.2	64.7 ± 6.8	30	30	17/13	15/15	Cryotherapy Compression device	Nothing	①②③⑧
Stocker et al^[13]^	2018	Switzerland	TKA	68.0 ± 8.6	73.1 ± 6.3	8	8	4/4	5/3	Cryotherapy Compression(cold packs) + CPM	Compression + CPM	①④⑥⑧
Wittig-Wells et al^[22]^	2015	American	TKA	64 ± 9.3	64 ± 9.3	15	14	6/9	5/9	Cryotherapy(cold packs)	Nothing	①⑤
Desteli et al^[23]^	2015	Turkey	TKA	65.14 ± 4.06	65.36 ± 6.98	42	45	22/20	22/23	Cryotherapy device	Nothing	①③⑦⑧⑨
Holm et al^[24]^	2012	Denmark	TKA	66 ± 12	67 ± 12	20	20	13/7	17/3	Cryotherapy(cold packs)	Nothing	①⑨
Radkowski et al^[25]^	2007	American	TKA	63.7 ± 10.4	66.9 ± 10.4	28	36	15/13	13/23	Cryotherapy Compression device	Compression	①②③④⑧
Kullenberg et al^[26]^	2006	Sweden	TKA	68.1 ± 6	68.9 ± 6.8	43	40	18/25	16/24	Cryotherapy Compression device	Nothing	①②③④⑧⑨
Holmström et al^[27]^	2005	Sweden	TKA	68	72	23	17	14/9	11/6	Cryotherapy Compression device	Nothing	①②③④⑥⑧
Morsi et al^[28]^	2002	Egypt	TKA	–	–	30	30	–	–	Cryotherapy Compression device + CPM	Compression + CPM	①②③④⑦⑧
Smith et al^[29]^	2002	Australia	TKA	72.1 ± 7.8	72 ± 7.1	44	40	21/23	21/19	Cryotherapy device	Nothing	①②③④⑥⑦⑧⑨
Gibbons et al^[30]^	2001	England	TKA	70	71	30	30	11/19	14/16	Cryotherapy Compression device	Compression	①②③④⑦⑧⑨
Webb et al^[31]^	1998	American	TKA	69	70.9	15	16	–	–	Cryotherapy Compression device	Nothing	①②③④⑦
Albrecht et al^[32]^	1997	Germany	TKA	71.9 73.8	69.6	32 35	31	–	–	Cryotherapy Compression device + CPM	CPM	①③⑧
Albrecht et al^[32]^	1997	Germany	THA	66 67.1	63.7	72 72	70	–	–	Cryotherapy Compression device + CPM	CPM	①③⑧
Ivey et al^[34]^	1994	American	TKA	64.5 ± 8.1 64.2 ± 10.3	66.9 ± 11.6	28 30	30	12/16 11/19	8/22	Cryotherapy Compression device	Nothing	⑧
Levy et al^[35]^	1993	American	TKA	74	73	40	40	7/33	8/32	Cryotherapy Compression device	Compression	①②③④⑧
Walker et al^[36]^	1991	American	TKA	75	70	15	15	–	–	Cryotherapy Compression device + CPM	CPM	②③⑧⑨
Leegwater et al^[37]^	2012	Netherlands	THA	66	68	12	14	–	–	Cryotherapy Compression device	Compression	②③⑧⑨
Saito et al^[38]^	2004	Japan	THA	59.3 ± 11.4	59.0 ± 11.2	22	23	–	–	Cryotherapy Compression device	Compression	①②③⑧

① Postoperative pain, ② analgesic use, ③ blood loss, ④ range of motion of joints, ⑤ patients satisfaction, ⑥ postoperative edema, ⑦ blood transfusion situation, ⑧ adverse events, ⑨ length of hospital stay, ⑩ time up and go test.

C = control group, CPM = continuous passive motion, CT = cryotherapy group.

### 3.3. Methodologic quality

Utilizing the Cochrane Risk of Bias 2.0 tool, we assessed the risk of bias in the 21 studies included in our analysis. The results of this evaluation are presented in Figures 2 and 3. The quality of the clinical studies varied, exhibiting a spectrum from low to high risk of bias. Specifically, 10 studies (47.6%) were identified as having a “high risk of bias,” 6 studies (28.6%) were categorized as having “some concerns,” and 5 studies (23.8%) were determined to have a “low risk of bias.”^[19,46]^

### 3.4. Results of the meta-analysis

The pooled outcomes are summarized in Table 2.

**Table 2:** Summary of pooled outcomes.

Outcomes	TKA							THA							Total						
	Studies	Participants	Result	Favours	95% CI	*P*	I^2^	Studies	Participants	Result	Favours	95% CI	*P*	I^2^	Studies	Participants	Result	Favours	95%CI	P	I^2^
Pain																					
POD 1 d	7	485	SMD = -0.13	Neither	-0.31 to 0.05	.17	58%	1	142	SMD = -0.69	Cryotherapy	-1.03 to -0.35	<.001	NA	7	627	SMD = -0.25	Cryotherapy	-0.42 to -0.09	0.002	68%
POD 2 d	4	293	SMD = -0.64	Cryotherapy	-0.88 to -0.4	<.001	24%	1	142	SMD = -0.69	Cryotherapy	-1.03 to -0.36	<.001	NA	4	435	SMD = -0.66	Cryotherapy	-0.86 to -0.46	<0.001	7%
POD 3 d	6	387	SMD = -0.14	Neither	-0.51 to 0.23	.45	67%	–	–	–	–	–	–	–	6	387	SMD = -0.14	Neither	-0.51 to 0.23	0.45	67%
POD 6 d	2	76	SMD = -1.11	Neither	-2.85 to 0.63	.21	89%	–	–	–	–	–	–	–	2	76	SMD = -1.11	Neither	-2.85 to 0.63	0.21	89%
POD 6 w	2	76	SMD = -0.16	Neither	-0.61 to 0.30	.50	0%	–	–	–	–	–	–	–	2	76	SMD = -0.16	Neither	-0.61 to 0.30	0.50	0%
Analgesic use	9	698	SMD = -0.48	Cryotherapy	-0.74 to -0.22	.0003	64%	2	93	SMD = -0.59	Neither	-1.49 to 0.31	.2	76%	11	791	SMD = -0.49	Cryotherapy	-0.74 to -0.25	<0.001	64%
Blood loss																					
HB change	4	310	SMD = -1.65	Cryotherapy	-2.65 to -0.66	.001	93%	–	–	–	–	–	–	–	4	310	SMD = -1.65	Cryotherapy	-2.65 to -0.66	0.001	93%
HB POD 2 d	2	171	SMD = 0.54	Neither	-0.52 to 1.60	.32	91%	–	–	–	–	–	–	–	2	171	SMD = 0.54	Neither	-0.52 to 1.60	0.32	91%
Blood loss from drainage	10	658	MD = -116.95	Cryotherapy	-194.38 to -39.51	.003	90%	1	26	MD = 24.00	Neither	-52.40 to 100.40	.54	NA	11	684	MD = -100.20	Cryotherapy	-171.74 to -28.65	0.006	89%
Extravasation of blood into soft tissue	2	140	MD = -410.48	Cryotherapy	-521.02 to 299.95	<.001	0%	–	–	–	–	–	–	–	2	140	MD = -410.48	Cryotherapy	-521.02 to -299.95	<0.001	0%
Total blood loss	3	200	MD = -408.66	Neither	-929.09 to 111.77	.12	98%	1	45	MD = -13.00	Neither	-350.17 to 324.17	.94	NA	4	245	MD = -318.05	Neither	-749.85 to 113.74	0.15	97%
ROM																					
ROM-POD 1 d	5	309	MD = 2.97	Neither	-4.25 to 10.19	.42	90%	1	142	MD = 12.2	noncryotherapy	11.26 to 13.14	<.001	NA	5	451	MD = 5.19	noncryotherapy	0.12 to 10.26	0.04	93%
ROM-POD 2 d	2	150	MD = 8.70	Neither	-11.39 to 28.79	.40	98%	1	142	MD = 15.80	noncryotherapy	14.73 to 18.61	<.001	NA	3	292	MD = 11.67	noncryotherapy	4.73 to 18.61	0.001	95%
ROM-POD 1 w	3	156	MD = 11.68	Non-cryotherapy	7.37 to 16.00	<.001	0%	–	–	–	–	–	–	–	3	156	MD = 11.68	noncryotherapy	7.37 to 16.00	<0.001	0%
ROM at discharge	2	183	MD = 6.35	Neither	-0.13 to 12.84	.05	57%	–	–	–	–	–	–	–	2	183	MD = 6.35	Neither	-0.13 to 12.84	0.05	57%
Patient satisfaction	2	130	MD = -0.12	Neither	-0.47 to 0.22	.48	34%	–	–	–	–	–	–	–	2	130	MD = -0.12	Neither	-0.47 to 0.22	0.48	34%
Edema																					
POD 1 d	2	104	SMD = -0.02	Neither	-0.40 to 0.37	.93	0%	–	–	–	–	–	–	–	2	104	SMD = -0.02	Neither	-0.40 to 0.37	0.93	0%
POD 1 w	2	76	SMD = 0.06	Neither	-0.95 to 1.07	.90	69%	–	–	–	–	–	–	–	2	76	SMD = 0.06	Neither	-0.95 to 1.07	0.90	69%
POD 6 w	2	116	SMD = 0.15	Neither	-0.65 to 0.95	.72	58%	–	–	–	–	–	–	–	2	116	SMD = 0.15	Neither	-0.65 to 0.95	0.72	58%
Transfusion rate	4	238	RR = 0.75	Neither	0.23 to 2.46	.63	73%	–	–	–	–	–	–	–	4	238	RR = 0.75	Neither	0.23 to 2.46	0.63	73%
Adverse events	14	920	RR = 0.99	Neither	0.68 to 1.44	.94	0%	2	72	RR = 1.08	Neither	0.15 to 7.57	.94	0%	16	992	RR = 0.99	Neither	0.68 to 1.44	0.96	0%
LOS	6	384	MD = 0.12	Neither	-0.71 to 0.94	.78	82%	1	26	MD = -0.25	Neither	-1.20 to 0.70	.61	NA	7	410	MD = 0.06	Neither	-0.65 to 0.77	0.86	79%

95% CI = 95% confidence interval, LOS = length of hospital stay, NA = not applicable.

#### 3.4.1. Postoperative pain

**Figure 4.**
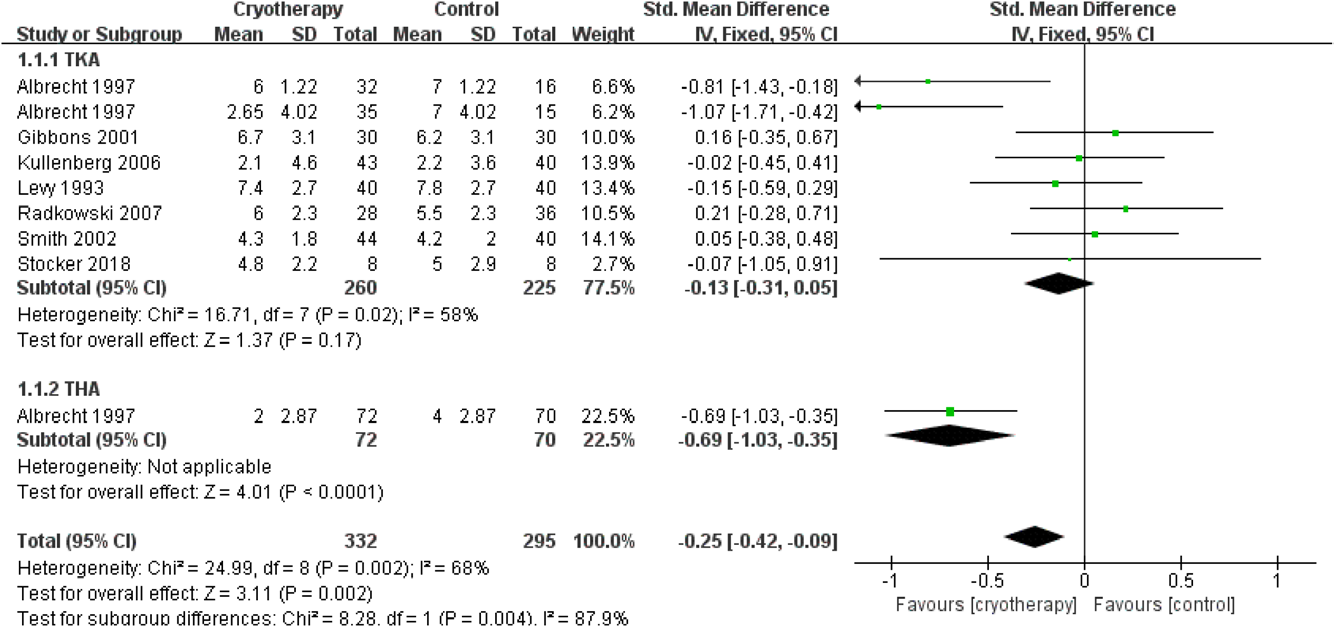
Effect of cryotherapy for postoperative pain on the 1st day versus control treatment.

**Figure 5.**
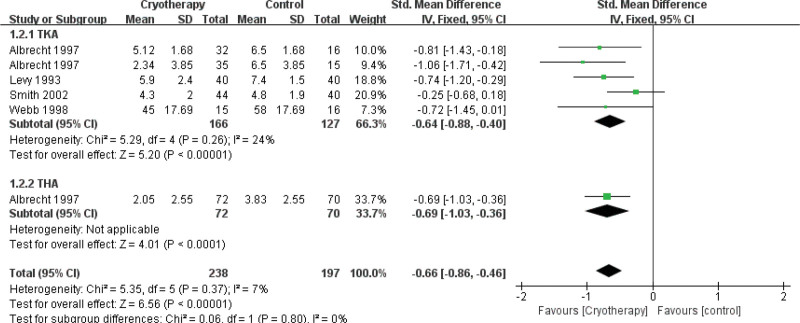
Effect of cryotherapy for postoperative pain on the 2nd day versus control treatment.

Changes in pain perception following cryotherapy versus non-cryotherapy were evaluated across 10 studies^[28,29,33,34,36–40,42]^ at multiple observation time points, with pain assessed using the Numerical Rating Scale or Visual Analogue Scale. Subgroup analyses stratified by joint replacement type were performed for the 1st^[29,33,34,37,38,40,42]^ and 2nd postoperative days (PODs)^[37,39,40,42]^ (Figs. 4 and 5, respectively). The analysis revealed that cryotherapy was associated with a significant improvement in pain for patients undergoing TKA and THA on the 2nd POD compared to the control group. In contrast, a significant reduction in pain on the 1st day was observed exclusively in THA patients. No significant pain reduction was associated with cryotherapy at the 3rd day,^[29,33,34,37,38,42]^ 6th day,^[29,36]^ or 6th week^[28,29]^ following TKA.

#### 3.4.2. Analgesic use

**Figure 6.**
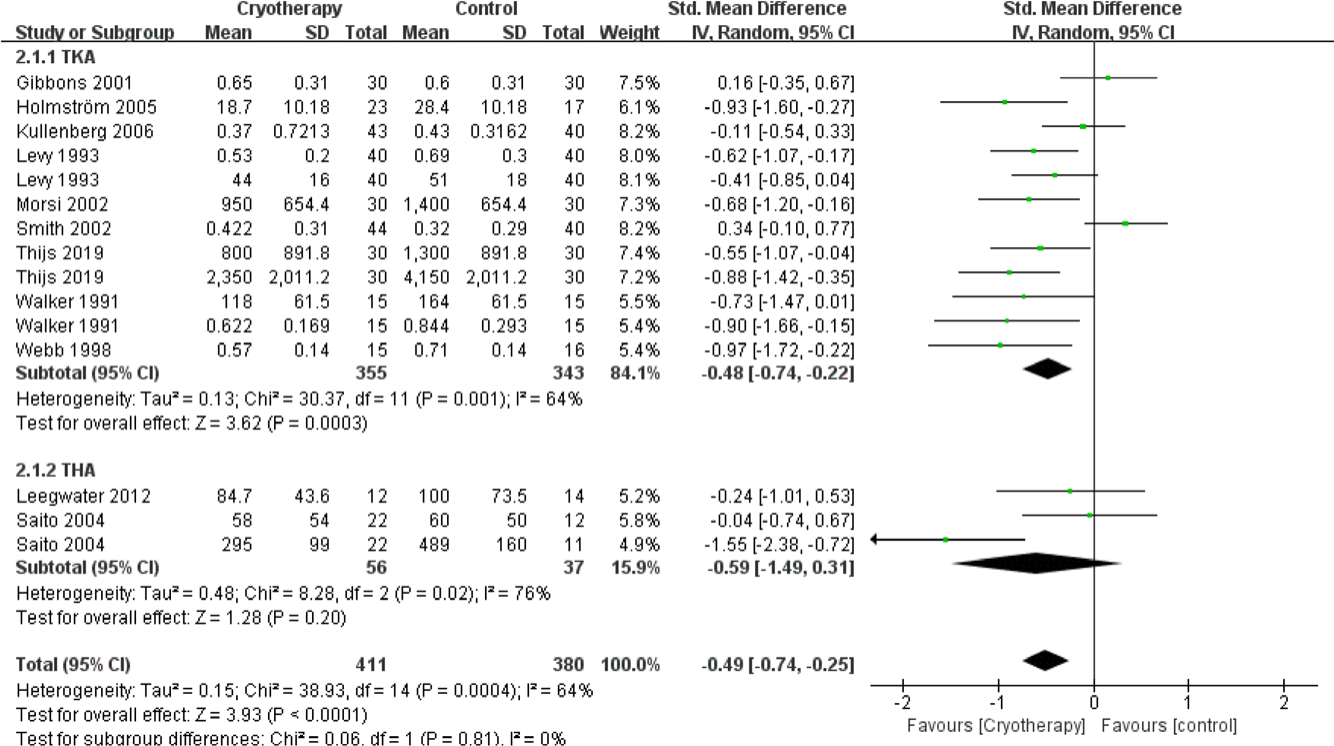
Effect of cryotherapy on analgesic use versus control treatment.

A pooled analysis of eleven studies^[28,34–39,42–45]^ compared postoperative analgesic requirements between cryotherapy and non-cryotherapy groups following TJA. The results indicated a significant reduction in analgesic use for TKA patients receiving cryotherapy. In contrast, cryotherapy failed to demonstrate a significant effect on analgesic consumption after THA (Fig. 6). Two studies^[26,33]^ were excluded from the analysis because the relevant data could not be extracted. With respect to opioid utilization, divergent findings were reported: Brouwers et al noted a significant reduction in the use of opioid escape medications during periods of severe pain in the cryotherapy group, while Radkowski et al^[33]^ observed no significant difference in overall postoperative opioid consumption between the groups.

#### 3.4.3. Blood loss

Postoperative blood loss was evaluated based on 2 parameters: the change in hemoglobin levels (mmol/L or g/L) and the quantified volume of blood loss (mL). The volumetric assessment comprised total blood loss, blood volume collected via drainage tubes, and blood loss into the subcutaneous soft tissue.

##### 3.4.3.1. Hemoglobin

**Figure 7.**
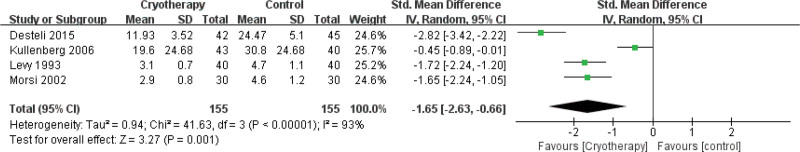
Effect of cryotherapy on hemoglobin change versus control treatment.

Four studies^[31,34,36,42]^ evaluated differences in hemoglobin change levels (mmol/L or g/L) following cryotherapy versus non-cryotherapy. The meta-analysis revealed a statistically significant advantage for cryotherapy in preserving hemoglobin change levels after TKA (Fig. 7). Conversely, 2 studies found no significant intergroup difference in hemoglobin levels (g/L) specifically on the 2nd POD.^[31,37]^ Furthermore, 1 study^[34]^ reported no significant effect on hemoglobin levels (mmol/L) on the 1st day after surgery, further supporting the time-dependent nature of the outcomes.

##### 3.4.3.2. Amount of blood loss

**Figure 8.**
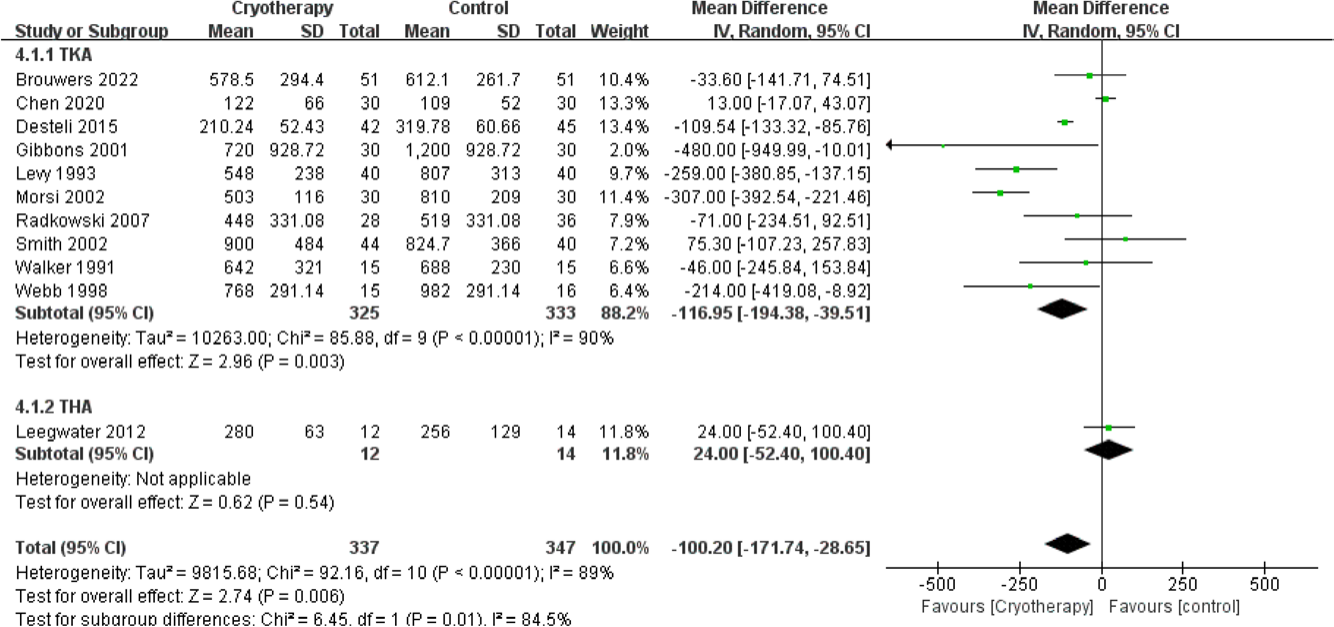
Effect of cryotherapy on blood loss from drainage versus control treatment.

**Figure 9.**
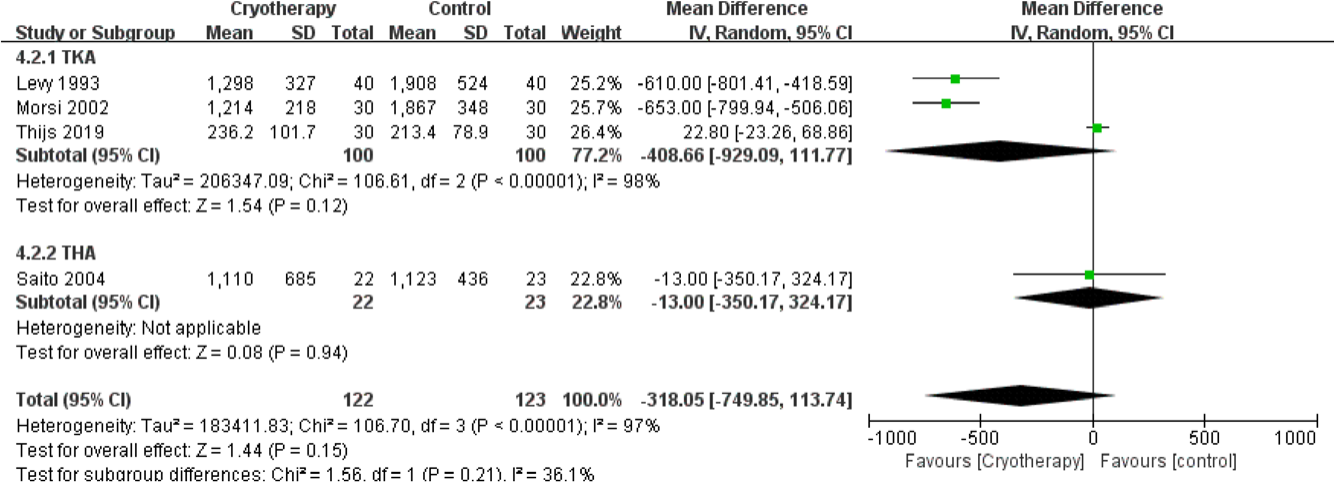
Effect of cryotherapy on total blood loss versus control treatment.

**Figure 10.**
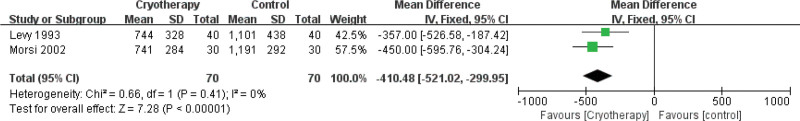
Effect of cryotherapy on extravasation of blood into soft tissue versus control treatment.

Eleven studies^[25,27,31,33,36–39,42–44]^ assessed the effect of cryotherapy on postoperative drainage volume. The meta-analysis revealed that cryotherapy significantly reduced drainage after TKA but not after THA (Fig. 8). Regarding total blood loss (mL), a separate analysis of 4 studies^[28,36,42,45]^ showed no significant benefit for cryotherapy in either TKA or THA (Fig. 9). Conversely, in the assessment of subcutaneous bleeding, 2 studies^[36,42]^ consistently found that cryotherapy was associated with a significant reduction in TKA patients (Fig. 10).

#### 3.4.4. ROM of joints

**Figure 11.**
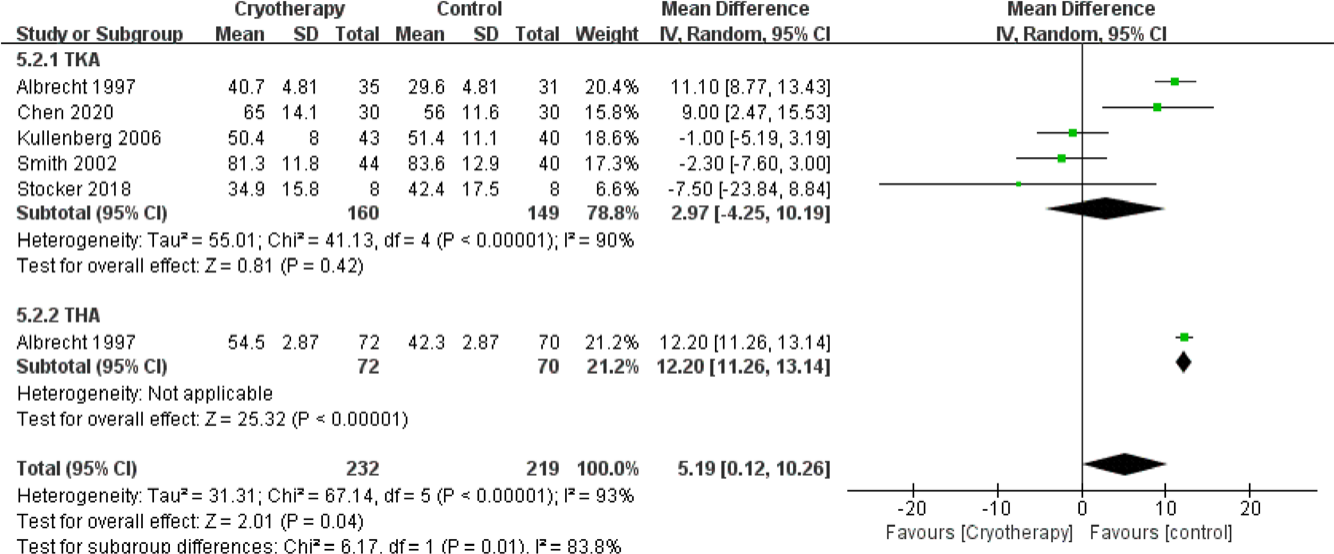
Effect of cryotherapy on range of motion-degrees of flexion POD 1 day versus control treatment. POD = postoperative day.

**Figure 12.**
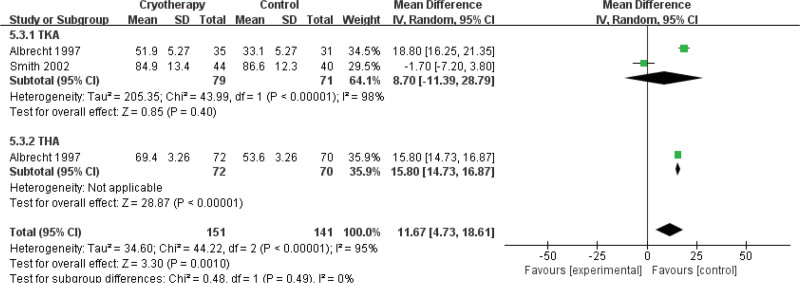
Effect of cryotherapy on range of motion-degrees of flexion POD 2 day versus control treatment. POD = postoperative day.

**Figure 13.**
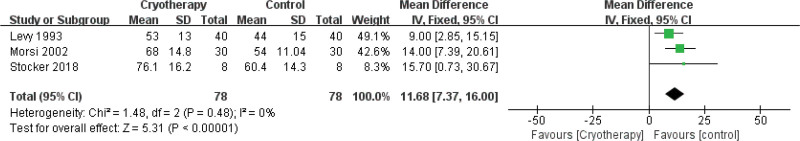
Effect of cryotherapy on range of motion-degrees of flexion POD 1 week versus control treatment. POD = postoperative day.

A systematic analysis of 8 comparative studies^[26,27,29,34,36,37,40,42]^ evaluated the impact of cryotherapy on joint flexion ROM in the early postoperative period following TKA and THA. Assessments were conducted at predefined intervals: POD1, POD2, 1 week after surgery, and pre-discharge. Subgroup analyses were stratified by procedure type (TKA vs THA) for POD1 and POD2. Five studies^[27,29,34,37,40]^ demonstrated no significant improvement in TKA flexion ROM with cryotherapy compared to non-cryotherapy controls on POD1. One study^[40]^ further indicated that cryotherapy following THA was associated with a reduction in joint flexion ROM at this time point (Fig. 11). A consistent pattern was observed on POD2, with 2 studies^[37,40]^ reporting no beneficial effect of cryotherapy on TKA flexion ROM and 1 study noting a decrement in ROM among THA recipients (Fig. 12). At the 1-week postoperative assessment, 3 studies^[29,36,42]^ identified a statistically significant reduction in TKA flexion ROM in the cryotherapy group relative to controls (Fig. 13). However, by the time of discharge,^[26,34]^ no significant intergroup difference in TKA flexion ROM was observed.

#### 3.4.5. Patients satisfaction

**Figure 14.**
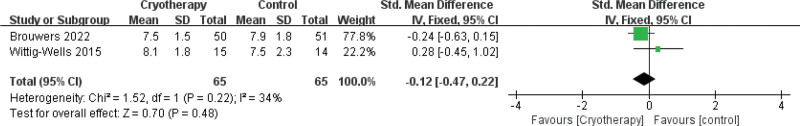
Effect of cryotherapy on patient satisfaction versus control treatment.

In 2 studies,^[25,30]^ patient satisfaction was reported to be comparable between the cryotherapy and non-cryotherapy groups, a finding demonstrated by the absence of statistically significant differences in satisfaction scores (Fig. 14).

#### 3.4.6. Postoperative edema

**Figure 15.**
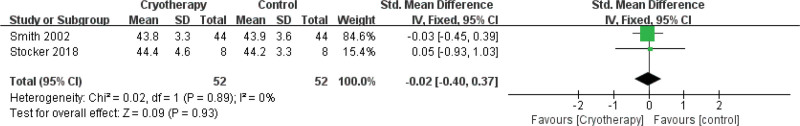
Effect of cryotherapy on knee circumference POD 1 day versus control treatment. POD = postoperative day.

**Figure 16.**
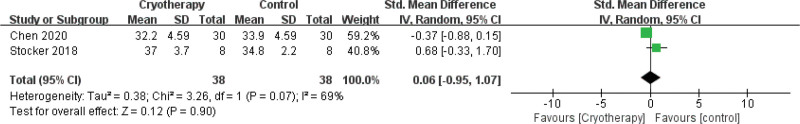
Effect of cryotherapy on knee circumference POD 1 week versus control treatment. POD = postoperative day.

**Figure 17.**
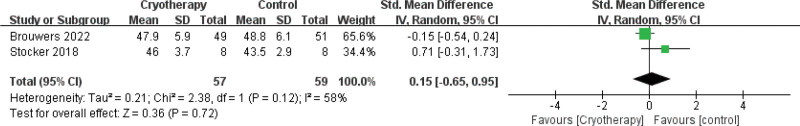
Effect of cryotherapy on knee circumference POD 6 week versus control treatment. POD = postoperative day.

Joint swelling was assessed by measuring the circumference (in centimeters) at a location 5 cm proximal to the knee joint. Although 6 trials^[25–27,29,35,37]^ reported this outcome, only 4 studies^[25,27,29,37]^ were incorporated into the meta-analysis. One study^[32]^ was excluded owing to incomplete data, and another^[26]^ provided swelling data based on lower limb volume calculations rather than direct circumferential measurements. Comparative analysis revealed that cryotherapy did not result in a statistically significant reduction in knee circumference relative to the non-cryotherapy group at POD 1^[29,37]^ (Fig. 15), postoperative week 1^[27,29]^ (Fig. 16), or postoperative week 6^[25,29]^(Fig. 17) following total knee arthroplasty.

#### 3.4.7. Transfusion

**Figure 18.**
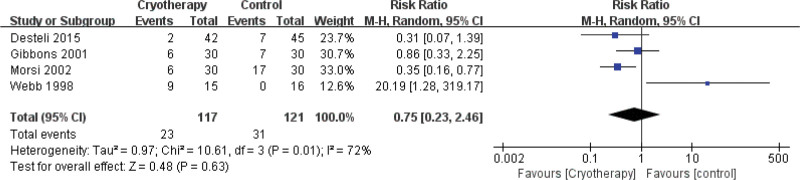
Effect of cryotherapy on transfusion rate versus control treatment.

Meta-analysis of 4 studies^[31,36,38,39]^ revealed no statistically significant difference in postoperative transfusion rates between patients receiving cryotherapy and those not receiving cryotherapy following TKA (Fig. 18). One study^[37]^ was excluded from this analysis as it reported total postoperative blood transfusion volume, an outcome measure inconsistent with the transfusion rates evaluated in the other included studies. That same study further indicated no advantage of cryotherapy over compression bandages in reducing postoperative blood transfusion.

#### 3.4.8. Adverse events

**Figure 19.**
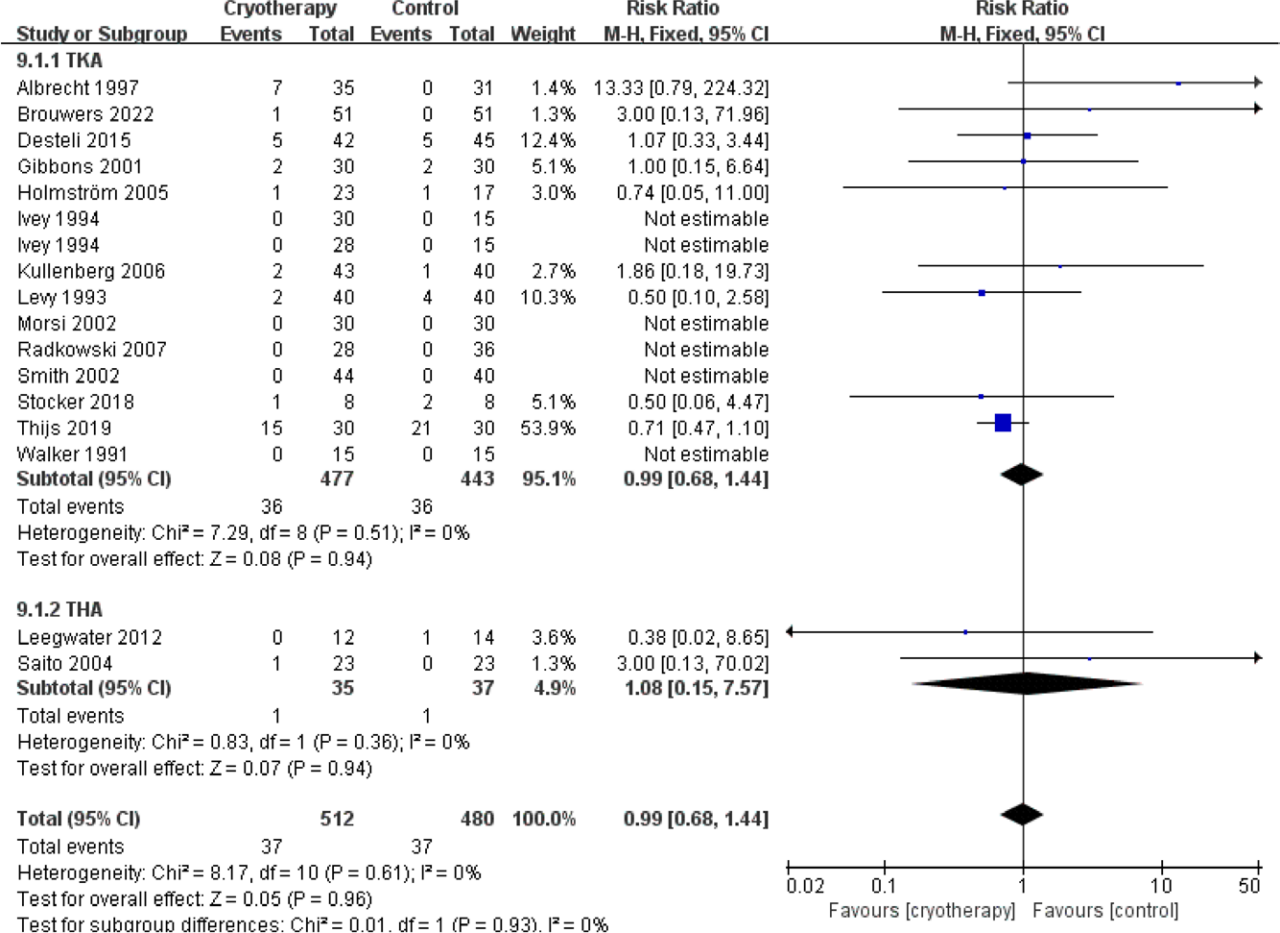
Effect of cryotherapy on adverse events versus control treatment.

The meta-analysis of 16 studies^[25,28,29,31,33–38,40–45]^ revealed no significant increase in the incidence of adverse events following total knee or hip arthroplasty with the use of cryotherapy compared to non-cryotherapy groups (Fig. 19).

#### 3.4.9. Length of hospital stay

**Figure 20.**
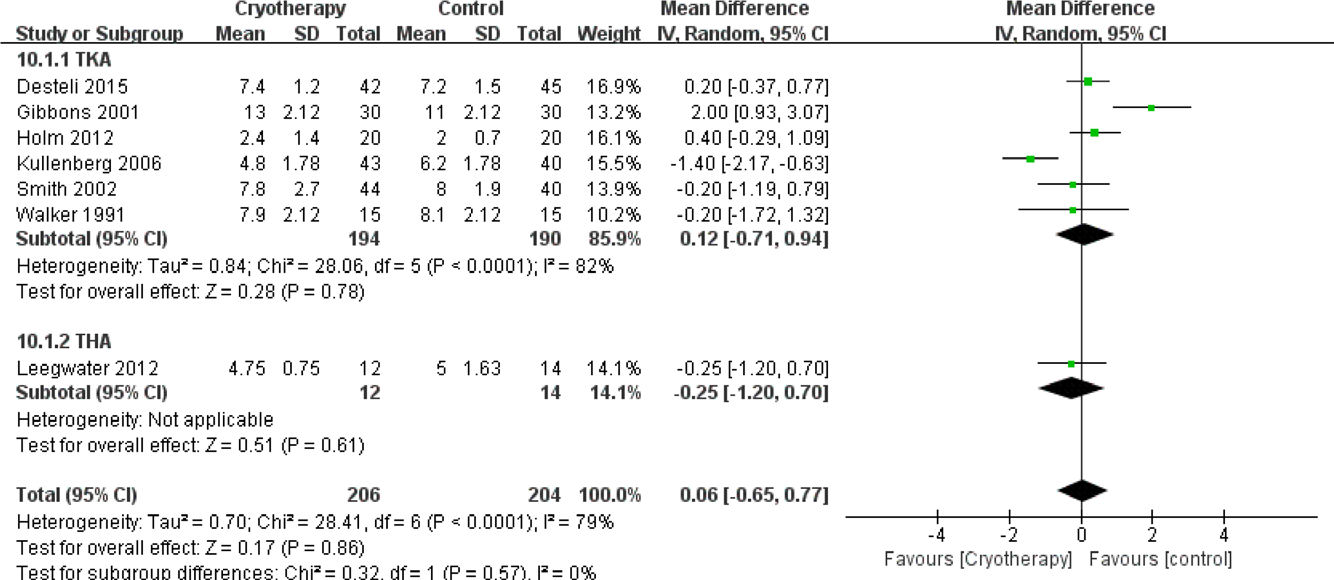
Effect of cryotherapy on length of hospital stay versus control treatment.

A comparative analysis of 7 studies^[31,32,34,37,38,43,44]^ demonstrated no statistically significant difference in the length of hospital stay between cryotherapy and non-cryotherapy groups following total knee or hip arthroplasty (Fig. 20).

### 3.5. Publication bias

**Figure 21.**
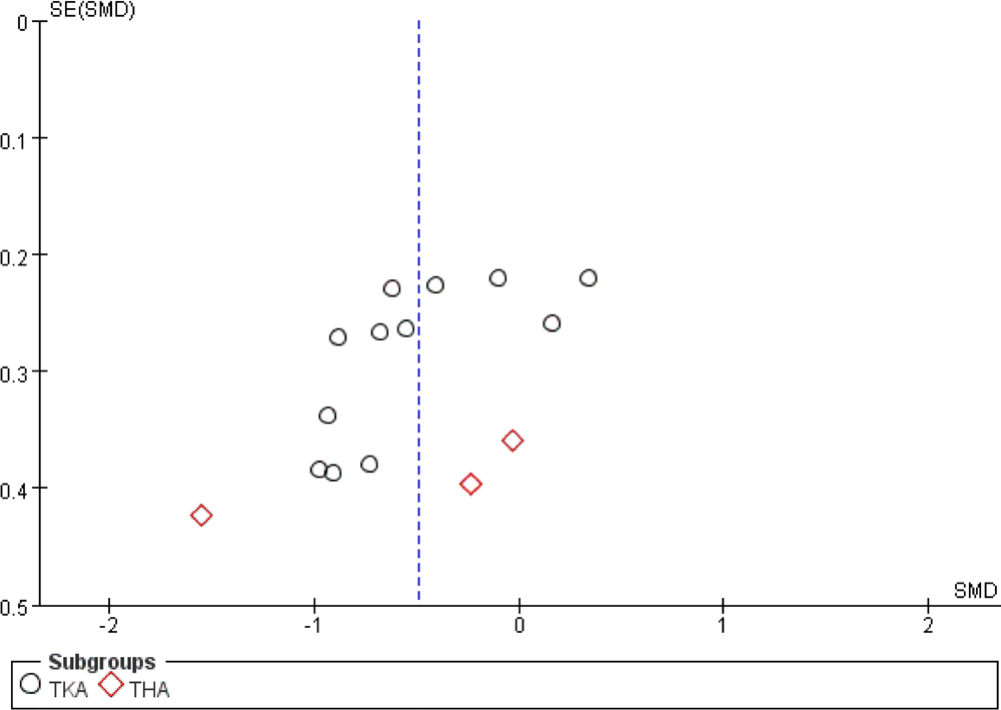
Funnel plot of comparison – cryotherapy on analgesic use versus control treatment.

**Figure 22.**
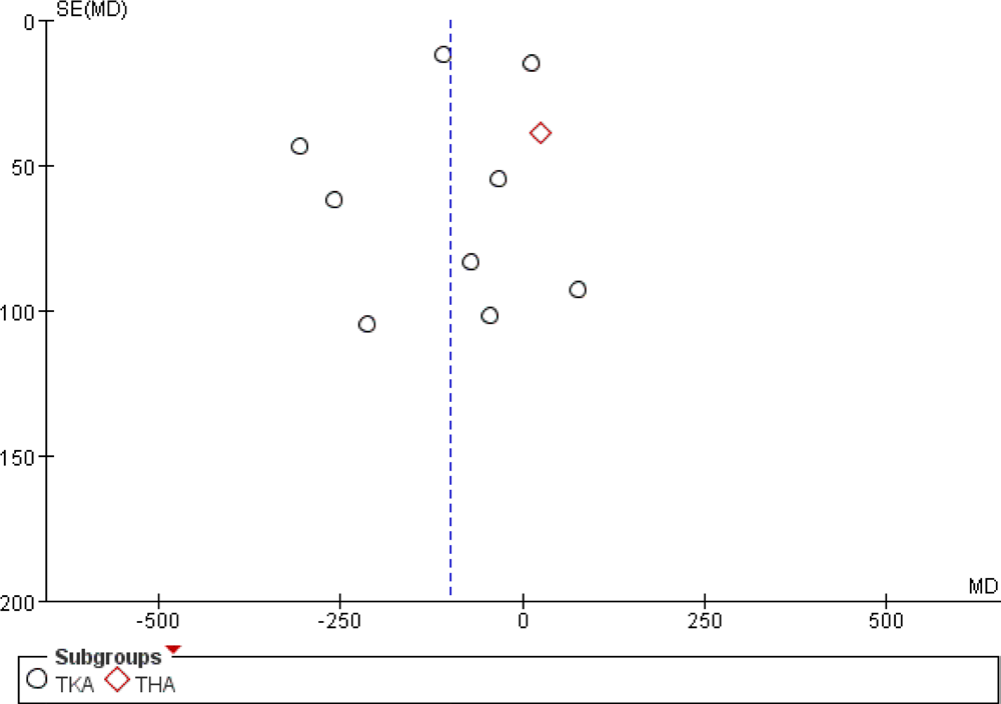
Funnel plot of comparison – cryotherapy on blood loss from drainage versus control treatment.

**Figure 23.**
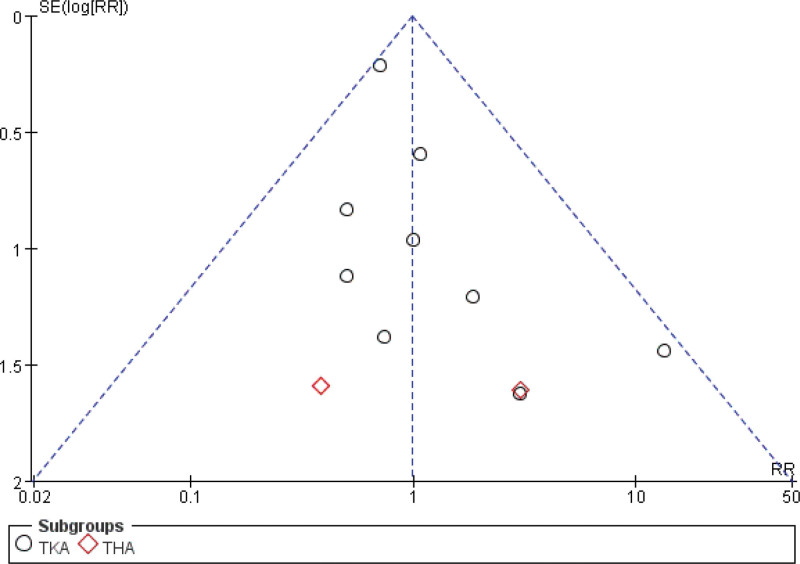
Funnel plot of comparison – cryotherapy on adverse events versus control treatment.

The funnel plots presented in the study indicate the presence of publication bias. Figures 21–23 illustrate 3 such funnel plots. An increased number of studies falling outside the 95% confidence interval suggests a higher degree of publication bias. Conversely, a more symmetrical distribution of studies on either side of the vertical line indicates a lower level of publication bias.

### 3.6. GRADE assessment

Several meta-analyses incorporated merely 2 studies, each with limited sample sizes. Consequently, to conduct a thorough and rigorous evaluation of the quality of outcome measures, we employed the GRADE tool to assess the quality of evidence for all outcome measures included in the analysis. The evidence quality for the outcomes was assessed as ranging from very low to moderate, primarily due to limitations such as risk of bias, imprecision, and heterogeneity. For pain scores, the certainty of evidence ranged from very low to moderate. The evidence for analgesic use and blood loss were rated as very low to low in quality. The quality of evidence pertaining to ROM, patient satisfaction, knee circumference, transfusion rate, and length of hospital stay were assessed as very low. The evidence for adverse events were rated as low in quality. Table 3 provides an overview of the GRADE evidence. The following 6 factors contribute to the serious hazards of deviations: the process report on the implementation of the random method is insufficient. Inadequate allocation concealment. Inadequate reporting on the implementation of blinding. There is a lot of study heterogeneity (*P* < .1, *I*^2^ > 50%). There are fewer than 400 participants in all the trials. The result is not significant.^[47]^ The above results’ evidence quality evaluation results can be used to guide clinical practice. To support the evidence of cryotherapy’s effectiveness in TJA, more large-sample, high-quality RCTs are required.^[24]^

**Table 3:** GRADE evidence profile.

Quality assessment							No. of patients		Effect		Quality	Importance
No. of studies	Design	Risk of bias	Inconsistency	Indirectness	Imprecision	Other considerations	Cryotherapy	Control	Relative (95% CI)	Absolute		
Pain POD 1 d (better indicated by lower values)												
7	Randomized trials	Serious*,†,‡	Serious§	No serious indirectness	No serious imprecision	None	332	295	-	SMD 0.25 lower (0.25 to 0.09 lower)	⊕⊕ΟΟ LOW	CRITICAL
Pain POD 1 d-TKA (better indicated by lower values)												
7	Randomized trials	Serious*,†,‡	Serious§	No serious indirectness	Serious‖	None	260	225	-	SMD 0.13 lower (0 to 0.05 higher)	⊕ΟΟΟ VERY LOW	CRITICAL
Pain POD 1 d-THA (better indicated by lower values)												
1	Randomized trials	Very serious*,†,‡	Very serious§	No serious indirectness	Serious¶	None	72	70	-	SMD 0.69 lower (0 higher to 0.35 lower)	⊕ΟΟΟ VERY LOW	CRITICAL
Pain POD 2 d (better indicated by lower values)												
5	Randomized trials	Serious*,†,‡	No serious inconsistency	No serious indirectness	No serious imprecision	None	238	197	-	MD 0.66 lower (0.77 to 0.46 lower)	⊕⊕⊕Ο MODERATE	CRITICAL
Pain POD 2 d-TKA (better indicated by lower values)												
4	Randomized trials	Serious*,†,‡	No serious inconsistency	No serious indirectness	Serious¶	None	166	127	-	SMD 0.64 lower (0 higher to 0.4 lower)	⊕⊕ΟΟ LOW	CRITICAL
Pain POD 2 d-THA (better indicated by lower values)												
1	randomized trials	Serious*,†,‡	No serious inconsistency	No serious indirectness	Serious¶	None	72	70	-	SMD 0.69 lower (0 higher to 0.36 lower)	⊕⊕ΟΟ LOW	CRITICAL
Pain POD 3 d (better indicated by lower values)												
6	Randomized trials	Very serious*,†,‡	Serious§	No serious indirectness	Very serious‖,¶	None	193	194	-	SMD 0.14 lower (0.51 lower to 0.23 higher)	⊕ΟΟΟ VERY LOW	CRITICAL
Pain POD 6 d (better indicated by lower values)												
2	Randomized trials	Serious*,†,‡	Very serious^4^	No serious indirectness	Very serious‖,¶	None	38	38	-	SMD 1.11 lower (2.85 lower to 0.63 higher)	⊕ΟΟΟ VERY LOW	CRITICAL
Pain POD 6 wk (better indicated by lower values)												
2	Randomized trials	Serious*,†,‡	No serious inconsistency	No serious indirectness	Very serious‖,¶	None	38	38	-	SMD 0.16 lower (0.61 lower to 0.3 higher)	⊕ΟΟΟ VERY LOW	CRITICAL
Analgesic use (better indicated by lower values)												
11	Randomized trials	Serious*,†,‡	Serious§	No serious indirectness	No serious imprecision	None	411	380	-	SMD 0.49 lower (0.74 to 0.25 lower)	⊕⊕ΟΟ LOW	CRITICAL
Analgesic use – TKA (better indicated by lower values)												
9	Randomized trials	Serious*,†,‡	Serious§	No serious indirectness	No serious imprecision	None	355	343	-	SMD 0.48 lower (0.74 to 0.22 lower)	⊕⊕ΟΟ LOW	CRITICAL
Analgesic use – THA (better indicated by lower values)												
2	Randomized trials	Serious*,†,‡	Serious§	No serious indirectness	Very serious‖,¶	None	56	37	-	SMD 0.59 lower (1.49 lower to 0.31 higher)	⊕ΟΟΟ VERY LOW	CRITICAL
Blood loss–hemoglobin change (better indicated by lower values)												
4	Randomized trials	Serious*,†,‡	No serious inconsistency	No serious indirectness	Serious‖	None	155	155	-	SMD 1.65 lower (1.99 to 0.66 lower)	⊕⊕ΟΟ LOW	CRITICAL
Blood loss–hemoglobin POD 2 d (better indicated by lower values)												
2	Randomized trials	Very serious*,†,‡	Very serious§	No serious indirectness	Very serious‖,¶	None	86	85	-	SMD 0.54 higher (0.52 lower to 1.6 higher)	⊕ΟΟΟ VERY LOW	CRITICAL
Blood loss from drainage (better indicated by lower values)												
11	Randomized trials	Serious*,†,‡	Very serious§	No serious indirectness	No serious imprecision	None	337	347	-	MD 100.2 lower (177.8 to 28.65 lower)	⊕ΟΟΟ VERY LOW	CRITICAL
Blood loss from drainage – TKA (better indicated by lower values)												
10	Randomized trials	Serious*,†,‡	Very serious§	No serious indirectness	No serious imprecision	None	325	333	-	MD 116.95 lower (197.41 to 39.51 lower)	⊕ΟΟΟ VERY LOW	CRITICAL
Blood loss from drainage – THA (better indicated by lower values)												
1	Randomized trials	Serious*,†,‡	No serious inconsistency	No serious indirectness	Very serious‖,¶	None	12	14	-	MD 24 higher (52.4 lower to 100.4 higher)	⊕ΟΟΟ VERY LOW	CRITICAL
Total blood loss (better indicated by lower values)												
4	Randomized trials	Very serious*,†,‡	Very serious§	No serious indirectness	Very serious‖,¶	None	122	123	-	MD 318.05 lower (749.85 lower to 113.74 higher)	⊕ΟΟΟ VERY LOW	CRITICAL
Total blood loss **–** TKA (better indicated by lower values)												
3	Randomized trials	Very serious*,†,‡	Very serious§	No serious indirectness	Very serious‖,¶	None	100	100	-	MD 408.66 lower (929.09 lower to 111.77 higher)	⊕ΟΟΟ VERY LOW	CRITICAL
Total blood loss – THA (better indicated by lower values)												
1	Randomized trials	Very serious*,†,‡	No serious inconsistency	No serious indirectness	Very serious‖,¶	None	22	23	-	MD 13 lower (350.17 lower to 324.17 higher)	⊕ΟΟΟ VERY LOW	CRITICAL
Blood loss–extravasation of blood into soft tissue (better indicated by lower values)												
2	Randomized trials	Very serious*,†,‡	No serious inconsistency	No serious indirectness	Serious‖	None	70	70	-	MD 410.48 lower (521.02 to 299.95 lower)	⊕ΟΟΟ VERY LOW	CRITICAL
Range of motion–degrees of flexion POD 1 d (better indicated by lower values)												
5	Randomized trials	Serious*,†,‡	Very serious§	No serious indirectness	No serious imprecision	None	232	219	-	MD 5.19 higher (4.99 lower to 10.26 higher)	⊕ΟΟΟ VERY LOW	IMPORTANT
Range of motion–degrees of flexion POD 1 d – TKA (better indicated by lower values)												
5	Randomized trials	Serious*,†,‡	Very serious§	No serious indirectness	Serious¶	None	160	149	-	MD 2.97 higher (0 to 10.19 higher)	⊕ΟΟΟ VERY LOW	IMPORTANT
Range of motion–degrees of flexion POD 1 d – THA (better indicated by lower values)												
1	Randomized trials	Serious*,†,‡	Serious§	No serious indirectness	Serious‖	None	72	70	-	MD 12.2 higher (0 to 13.14 higher)	⊕ΟΟΟ VERY LOW	IMPORTANT
Range of motion–degrees of flexion POD 2 d (better indicated by lower values)												
2	Randomized trials	Serious*,†,‡	Serious§	No serious indirectness	Very serious‖,¶	None	151	141	-	MD 11.67 higher (0 to 18.61 higher)	⊕ΟΟΟ VERY LOW	IMPORTANT
Range of motion–degrees of flexion POD 2 d – TKA (better indicated by lower values)												
2	Randomized trials	Serious*,†,‡	Serious§	No serious indirectness	Very serious‖,¶	None	79	71	-	MD 8.7 higher (0 to 28.79 higher)	⊕ΟΟΟ VERY LOW	IMPORTANT
Range of motion–degrees of flexion POD 2 d – THA (better indicated by lower values)												
1	Randomized trials	Serious*,†,‡	Serious§	No serious indirectness	Serious‖	None	72	70	-	MD 15.8 higher (0 to 16.87 higher)	⊕ΟΟΟ VERY LOW	IMPORTANT
Range of motion–degrees of flexion POD 1 wk (better indicated by lower values)												
3	Randomized trials	Very serious*,†,‡	No serious inconsistency	No serious indirectness	Serious‖	None	78	78	-	MD 11.68 higher (7.37 to 16 higher)	⊕ΟΟΟ VERY LOW	IMPORTANT
Range of motion–degrees of flexion at discharge (better indicated by lower values)												
2	Randomized trials	Serious*,†,‡	Serious§	No serious indirectness	Very serious‖,¶	None	76	107	-	MD 6.35 higher (0.13 lower to 12.84 higher)	⊕ΟΟΟ VERY LOW	IMPORTANT
Patient satisfaction (better indicated by lower values)												
2	Randomized trials	Serious*,†,‡	No serious inconsistency	No serious indirectness	Very serious‖,¶	None	65	65	-	SMD 0.12 lower (0.47 lower to 0.22 higher)	⊕ΟΟΟ VERY LOW	NOT IMPORTANT
Knee circumference POD 1 d (better indicated by lower values)												
2	Randomized trials	Serious*,†,‡	No serious inconsistency	no serious indirectness	Very serious‖,¶	None	52	52	-	SMD 0.02 lower (0.4 lower to 0.37 higher)	⊕ΟΟΟ VERY LOW	NOT IMPORTANT
Knee circumference POD 1 wk (better indicated by lower values)												
2	Randomized trials	No serious risk of bias	Serious§	No serious indirectness	Very serious‖,¶	None	38	38	-	SMD 0.06 higher (0.95 lower to 1.07 higher)	⊕ΟΟΟ VERY LOW	NOT IMPORTANT
Knee circumference POD 6 wk (better indicated by lower values)												
2	Randomized trials	Serious*,†,‡	Serious§	No serious indirectness	Very serious‖,¶	None	57	59	-	SMD 0.15 higher (0.65 lower to 0.95 higher)	⊕ΟΟΟ VERY LOW	NOT IMPORTANT
Transfusion rate												
4	Randomized trials	Very serious*,†,‡	Serious§	No serious indirectness	Very serious‖,¶	None	23/117 (19.7%)	31/121 (25.6%)	RR 0.75 (0.23–2.46)	64 fewer per 1000 (from 197 fewer to 374 more)	⊕ΟΟΟ VERY LOW	NOT IMPORTANT
								19.4%		48 fewer per 1000 (from 149 fewer to 283 more)		
Adverse events												
16	Randomized trials	Serious*,†,‡	No serious inconsistency	No serious indirectness	Serious¶	None	37/512 (7.2%)	37/480 (7.7%)	RR 0.99 (0.68–1.44)	1 fewer per 1000 (from 25 fewer to 34 more)	⊕⊕ΟΟ LOW	IMPORTANT
								0%				
Adverse events – TKA												
15	Randomized trials	Serious*,†,‡	No serious inconsistency	No serious indirectness	Serious¶	None	36/477 (7.5%)	36/443 (8.1%)	RR 0.99 (0.68–1.44)	1 fewer per 1000 (from 26 fewer to 36 more)	⊕⊕ΟΟ LOW	IMPORTANT
								0%				
Adverse events – THA												
2	Randomized trials	Serious*,†,‡	No serious inconsistency	No serious indirectness	Serious‖,¶	None	1/35 (2.9%)	1/37 (2.7%)	RR 1.08 (0.15–7.57)	2 more per 1000 (from 23 fewer to 178 more)	⊕⊕ΟΟ LOW	IMPORTANT
								3.6%				
Length of hospital stay (better indicated by lower values)												
7	Randomized trials	Very serious*,†,‡	Very serious§	No serious indirectness	Serious‖	None	206	204	-	MD 0.06 higher (0.65 lower to 0.77 higher)	⊕ΟΟΟ VERY LOW	NOT IMPORTANT
Length of hospital stay – TKA (better indicated by lower values)												
6	Randomized trials	Very serious*,†,‡	Very serious§	No serious indirectness	Very serious‖,¶	None	194	190	-	MD 0.12 higher (0.71 lower to 0.94 higher)	⊕ΟΟΟ VERY LOW	NOT IMPORTANT
Length of hospital stay – THA (better indicated by lower values)												
1	Randomized trials	Very serious*,†,‡	No serious inconsistency	No serious indirectness	Very serious‖,¶	None	12	14	-	MD 0.25 lower (1.2 lower to 0.7 higher)	⊕ΟΟΟ VERY LOW	NOT IMPORTANT

RR = risk ratios, SMD = standardized mean difference, THA = total hip arthroplasty, TKA = total knee arthroplasty.

* Inadequate allocation concealment.

† The process report on the implementation of the random method is insufficient.

‡ Inadequate reporting on the implementation of blinding.

§ The heterogeneity between studies is large (*P* < .1, *I*^2^ > 50%).

‖ The total sample size of the studies is <400.

¶ The result is not significant.

## 4. Discussion

### 4.1. Effectiveness

This study leverages recent advancements in cryotherapy research related to TJA to evaluate the comprehensive effects of cryotherapy versus the absence of cryotherapy on various postoperative outcomes, including pain management, analgesic consumption, postoperative bleeding, joint ROM, patient satisfaction, joint swelling, transfusion rates, incidence of adverse events, and length of hospital stay in patients undergoing TKA and THA. Additionally, the study investigates the role of cryotherapy in facilitating joint rehabilitation. Subgroup analyses stratified by surgical type were performed within the meta-analysis to assess outcomes such as postoperative pain, analgesic consumption, postoperative blood loss, joint flexion, adverse event incidence, and length of hospital stay. Reports on outcomes such as the reduction of postoperative joint swelling, transfusion rates, and patient satisfaction were exclusively available for TKA, with no corresponding data for THA. The findings reveal inconsistent rehabilitation outcomes between cryotherapy applications in TKA and THA. Following TKA, cryotherapy has been shown to reduce postoperative pain on the 2nd day, decrease the required dosage of analgesics, minimize the extent of hemoglobin fluctuations, and reduce both subcutaneous soft tissue bleeding and drainage tube output when compared to patients not receiving cryotherapy. However, it also results in a decreased range of knee flexion during the 1st postoperative week. In patients undergoing THA, cryotherapy similarly reduces pain on the 1st and 2nd PODs but is associated with decreased joint flexion on both days. Notably, cryotherapy does not significantly impact the reduction of analgesic usage, drainage volume, or total fluid output following THA. When compared to patients who did not receive cold therapy-assisted joint rehabilitation, the use of cryotherapy does not increase the incidence of adverse reactions following either TKA or THA. Nevertheless, it does not enhance patient rehabilitation satisfaction, reduce joint swelling, lower transfusion rates, or shorten hospital stays.

In the context of joint rehabilitation, the analgesic efficacy of cryotherapy constitutes a critical outcome measure in this systematic review. Effective pain management not only reduces analgesic requirements but also enables earlier and more aggressive mobilization (a benefit corroborated by established evidence of cryotherapy in arthroscopic ligament reconstruction).^[48,49]^ Our analysis demonstrated clinically meaningful pain reduction on POD 2 following TKA and during the 1st 2 days after THA. However, these analgesic effects were not sustained at subsequent rehabilitation phases, including POD 3, day 6, and the 6-week functional training period. These temporal patterns align with Muaddi et al,^[50]^ who documented significant pain reduction limited to the initial PODs, and are consistent with other meta-analyses.^[24]^ The neurophysiological mechanism likely involves cryotherapy-mediated elevation of pain thresholds and endogenous endorphin release, which collectively alleviate pain from protective muscle spasms secondary to surgical trauma, thereby facilitating active participation in prescribed rehabilitation exercises.^[20]^ Importantly, the therapeutic window of cryotherapy appears limited to the acute inflammatory phase, with diminishing returns as patients transition to intensive functional training. While our evaluation spanned multiple rehabilitation milestones, the scarcity of high-quality studies at each timepoint introduces uncertainty in outcome interpretation, reflected in the GRADE assessment rating of very low to moderate evidence quality. Consequently, cryotherapy should be strategically deployed as an early-phase adjunct in TJA rehabilitation protocols, primarily during the immediate postoperative period to support initial mobility training, rather than as a sustained intervention throughout the rehabilitation continuum.

Our meta-analysis indicates that cryotherapy significantly reduces analgesic consumption following TKA, while showing no significant effect after THA. This result is consistent with previous systematic reviews^[50]^ and supported by Wyatt et al,^[51]^ though it conflicts with findings from Radkowski et al^[33]^ and Tedesco et al^[9]^ The analgesic mechanism of cryotherapy may involve reduced nerve conduction velocity and elevated pain threshold.^[52]^ However, Thienpont et al^[53]^ reported potential pain recurrence after cryotherapy discontinuation, suggesting its analgesic effects may be temporary. Analgesic consumption is directly influenced by pain management protocols, and variations in these protocols may explain the inconsistent findings regarding cryotherapy’s effect on analgesic use.^[25]^ The GRADE assessment revealed very low to low quality evidence for outcomes related to analgesic consumption, indicating limited reliability of current findings. Future studies should standardize pain management protocols when evaluating cryotherapy’s impact on analgesic use after TJA. Considering the adverse effects of analgesics (including gastrointestinal discomfort, drowsiness, and sedation^[31]^) which may hinder rehabilitation, cryotherapy represents a promising non-pharmacological approach to reduce analgesic requirements following joint replacement surgery.

Postoperative bleeding represents a critical determinant of recovery following joint replacement surgery, with potential to significantly impede rehabilitation progress. Excessive bleeding may lead to complications such as hematoma formation, joint fibrosis, and increased transfusion requirements, collectively prolonging hospitalization, delaying functional recovery, and elevating healthcare costs.^[17,54,55]^ The proposed mechanism of cryotherapy involves activation of cutaneous cold receptors, triggering sympathetic-mediated vasoconstriction that reduces local blood flow and promotes coagulation.^[56]^ In clinical orthopedic practice, postoperative bleeding is typically monitored through serial hemoglobin measurements, drainage output volume, and assessment of wound seepage. A comprehensive evaluation further incorporates total blood loss calculations, accounting for both visible absorption in surgical dressings and estimated occult bleeding. This systematic review provides an integrated analysis of these evidence-based bleeding outcome measures. Evidence indicates that cryotherapy reduces postoperative hemoglobin fluctuations, decreases drainage output, and limits subcutaneous hemorrhage after TKA, though it does not significantly affect total drainage volume. Postoperative blood loss following TJA is influenced by multiple factors; however, randomization in the included studies controlled for confounders such as surgical technique and coagulation status. Cryotherapy may reduce bleeding through cold-induced vasoconstriction while maintaining anticoagulation safety.^[31]^ Combined cryo-compression therapy further reduces hematoma formation,^[34]^ suggesting potential synergistic effects. These findings suggest cryotherapy primarily affects localized bleeding rather than total blood loss. This effect was not observed in THA, possibly due to anatomical differences, tourniquet use in TKA,^[57]^ and distinct occult blood loss mechanisms.^[58]^ However, evidence regarding cryotherapy in THA remains limited, with GRADE assessments indicating very low to low quality for bleeding-related outcomes.

ROM is a critical indicator for evaluating functional recovery after TJA. Cryotherapy, while not directly improving ROM, may aid rehabilitation by reducing pain and swelling. However, our meta-analysis found that cryotherapy was associated with reduced flexion angles at 1 week after TKA and during the 1st 1 to 2 days after THA. These results are consistent with Wyatt et al,^[51]^ who reported minimal effect of cryotherapy on ROM following TKA. The observed heterogeneity in study results may be due to the indirect and variable effect of cryotherapy on postoperative ROM, which is mediated by its impact on pain and swelling. In contrast, Zhou et al^[56]^ demonstrated that alternating cold and heat therapy significantly improved ROM after TKA, which was supported by Wilk et al^[59]^ in orthopedic rehabilitation settings. Therefore, alternating cold–heat therapy may provide superior benefits for joint functional recovery compared with cryotherapy alone.

Cryotherapy mitigates tissue swelling by reducing vascular permeability and fluid leakage.^[56]^ In this study, swelling was assessed through knee circumference measurements. No statistically significant differences in knee circumference were observed between the groups on POD 1, at week 1, or at week 6. Our results are consistent with the findings of Wyatt PB et al,^[51]^ who also reported that cryotherapy provided no sustained benefit in reducing swelling. Discrepancies in the anatomic reference points for circumference measurement^[25,29]^ and the formula used to compute swelling^[26]^ may have influenced these outcomes. Therefore, we conclude that cryotherapy did not demonstrate a significant effect on swelling or knee circumference compared with the non-cryotherapy group.

The meta-analysis revealed that cryotherapy did not have a statistically significant impact on patient satisfaction. This finding contrasts with previous research, such as study,^[60]^ which reported enhanced patient satisfaction following knee arthroscopy. Given that patient satisfaction is closely associated with the management of symptoms like postoperative pain and edema, the neutral overall outcome in our study may be attributed to the limited efficacy of cryotherapy in alleviating these symptoms. Specifically, cryotherapy demonstrated a significant effect on pain only during PODs 1 and 2 following TJA, with no statistically significant benefits observed at most other time points for either pain or edema. Additionally, potential heterogeneity among the populations in the included studies, such as study,^[60]^ may have influenced the results. In relation to postoperative blood transfusion, our meta-analysis demonstrates that cryotherapy does not exert a statistically significant impact on overall outcomes. It is well-established that joint replacement surgery is frequently associated with substantial postoperative blood loss.^[61]^ In instances of excessive hemorrhage, transfusion therapy becomes necessary, which can impede the accelerated recovery of patients undergoing joint replacement. This study identified that cryotherapy effectively reduces bleeding from postoperative drainage tubes and subcutaneous soft tissue hemorrhage, yet there is no significant difference in transfusion rates between the groups. Nonetheless, the findings are subject to potential bias due to low treatment doses and an insufficient number of studies. Consequently, further research is warranted to substantiate the efficacy of cryotherapy. In terms of hospital length of stay, our study revealed a non-statistically significant overall effect of cryotherapy. This finding aligns with previous literature,^[50]^ which similarly concluded that cryotherapy does not influence the length of stay. However, it is important to note that the population in the prior study primarily comprised postoperative patients, introducing a degree of heterogeneity when compared to our study population. Additionally, considering that hospital length of stay is affected by numerous confounding variables, it remains uncertain whether cryotherapy impacts this outcome within our specific cohort of joint replacement patients.

### 4.2. Safety

In our study,we focused on evaluating whether cryotherapy increases the incidence of safety-related adverse events. These events included postoperative nausea and vomiting, venous thromboembolism, wound complications (such as hematoma or persistent leakage), periprosthetic joint infection, prosthesis loosening, patient-reported discomfort, local skin reactions, infections, and cold-induced injury.^[16,17]^ Our meta-analysis revealed no statistically significant overall effect of cryotherapy compared to no cryotherapy. This finding is consistent with a previous study^[62]^ that compared continuous and traditional cryotherapy regimens. Additionally, funnel plot analysis demonstrated no substantial publication bias among the included studies, as indicated by the largely symmetrical distribution of points. Based on the available evidence, we conclude that cryotherapy appears to be safe following joint arthroplasty.

### 4.3. Strengths of this study

This review was conducted in accordance with a comprehensive, pre-established protocol. To the best of our knowledge, this meta-analysis incorporates findings from all RCTs that have investigated both control and cryotherapy groups to date, providing a thorough overview of the cumulative effects. Concurrently, outcome indicators were enhanced, and subgroup analyses were performed based on various types of joint replacement surgeries. Specifically, variations in hemoglobin levels and total blood loss were examined within the context of blood loss. Hemoglobin variations were further categorized into hemoglobin levels on the 1st POD and subsequent changes. Outcome measures such as total blood loss, blood loss into subcutaneous tissue, and drainage tube blood loss were assessed. Additionally, similar outcome measures, including postoperative pain, range of joint motion, and postoperative swelling, were analyzed. These aspects are not addressed in other comparable studies.^[50,62,63]^

### 4.4. Limitations of the study

This study is subject to several limitations. Firstly, the restricted inclusion of outcome measures across the studies considered prevented the analysis of all outcomes in subgroups based on the type of joint replacement surgery. Secondly, the analysis in this study was predicated on the observed similarity in efficacy between cold therapy machines and cold therapy packs. However, due to the limited number of studies involving cold packs and the associated outcome measures, it was not feasible to conduct subgroup analyses for different forms of cryotherapy. As research in this area advances and the number of relevant studies increases, future investigations may be able to more thoroughly compare the efficacy of various cryotherapy modalities. Thirdly, the control group did not encompass other non-cryotherapy interventions, as it was limited to no intervention, compression therapy, and CPM groups. Fourth, the cryotherapy protocols lacked standardization concerning cooling temperature, exposure duration, and minimum temperature thresholds. All variables were synthesized based on the values reported in the included studies. Fifth, addressing publication bias and potential biases in study assessments continues to pose significant challenges. Lastly, the retrieval of gray literature was not possible during the literature search due to limited access to pertinent databases.^[64]^

## 5. Conclusion

This meta-analysis indicates that cryotherapy, when compared to the absence of such intervention, can facilitate joint rehabilitation following joint replacement surgery. However, it is essential to consider the specific type of joint replacement surgery undertaken by the patient. Our findings reveal that the rehabilitative effects of cryotherapy differ between TKA and THA. In the context of TKA, cryotherapy has been shown to reduce postoperative pain on the 2nd day, decrease the need for analgesic medication, and minimize postoperative blood loss by stabilizing hemoglobin levels, reducing blood infiltration into subcutaneous soft tissues, and decreasing drainage tube output. Conversely, in the THA subgroup analysis, cryotherapy was effective in alleviating pain on the 1st and 2nd PODs but did not demonstrate a significant impact on analgesic consumption or blood loss reduction. Cryotherapy did not elevate the risk of adverse events. Nevertheless, it neither enhanced patient satisfaction with recovery nor reduced transfusion rates or shortened hospital stays. Importantly, cryotherapy did not improve joint ROM as initially expected. Subgroup analysis revealed that cryotherapy was not advantageous in achieving greater flexion angles during joint rehabilitation following THA.

Despite employing the GRADE approach to evaluate all outcome measures, the quality of evidence varied from very low to moderate, primarily due to imbalances in risk of bias, accuracy, and heterogeneity among the studies. Consequently, the design and execution of more rigorous, large-sample, high-quality multicenter RCTs are necessary to provide robust evidence supporting the efficacy of cold therapy in promoting joint rehabilitation following TJA. Additionally, it is essential to identify appropriate application scenarios for cold therapy based on its clinical objectives and the primary needs of patients. When TJA patients utilize cold therapy as an adjunctive tool for postoperative joint rehabilitation and functional exercise, the findings of this study can inform clinical decision-making.

## Author contributions

**Conceptualization:** Yu Xie.

**Data curation:** Lijun Wang, Shizheng Du, Xin Li.

**Formal analysis:** Yu Xie, Xin Li.

**Methodology:** Yu Xie, Shizheng Du.

**Software:** Yu Xie, Shizheng Du, Xin Li.

**Supervision:** Weiyu Pan, Junjuan Zhang.

**Visualization:** Yu Xie.

**Writing – original draft:** Yu Xie.

**Writing – review & editing:** Yu Xie, Weiyu Pan.

## Supplementary Material


