# Supplementary material for: Cryotherapy in joint arthroplasty rehabilitation: Effects on pain, analgesic consumption, blood loss, and range of motion
Source: Medicine (Baltimore). 2026 Jan 2;105(1):e46802. doi: 10.1097/MD.0000000000046802 (PMC12778110; doi:10.1097/MD.0000000000046802)
Supplement: Supplementary file 1 [file medi-105-e46802-s001.docx]

| 1. Pubmed | |
| --- | --- |
| #1 | Search "Arthroplasty, Replacement, Ankle"[Mesh] OR "Arthroplasty, Replacement, Elbow"[Mesh] OR "Arthroplasty, Replacement, Finger"[Mesh] OR "Arthroplasty, Replacement, Knee"[Mesh] OR "Arthroplasty, Replacement, Hip"[Mesh] OR "Arthroplasty, Replacement"[Mesh] OR "Arthroplasty, Replacement, Shoulder"[Mesh] |
| #2 | Search: ("Elbow Prosthesis"[Mesh]) OR ( "Knee Prosthesis"[Mesh] OR "Joint Prosthesis"[Mesh] OR "Hip Prosthesis"[Mesh] OR "Shoulder Prosthesis"[Mesh] ) |
| #3 | Search: (((((((((((((arthroplast*[Title/Abstract]) OR (replac*[Title/Abstract])) OR (knee arthroplast*[Title/Abstract])) OR (knee replac*[Title/Abstract])) OR (hip replac*[Title/Abstract])) OR (hip arthroplast*[Title/Abstract])) OR (shoulder arthroplast*[Title/Abstract])) OR (shoulder replac*[Title/Abstract])) OR (elbow arthroplast*[Title/Abstract])) OR (elbow replac*[Title/Abstract])) OR (ankle replac*[Title/Abstract])) OR (ankle arthroplast*[Title/Abstract])) OR (finger arthroplast*[Title/Abstract])) OR (finger replac*[Title/Abstract])) OR (joint arthroplast*[Title/Abstract])) OR (joint replac*[Title/Abstract])) OR (replacement arthroplast*[Title/Abstract])) |
| #4 | (((((((prosthe*[Title/Abstract]) OR (knee prosthe*[Title/Abstract])) OR (hip prosthe*[Title/Abstract])) OR (shoulder prosthe*[Title/Abstract])) OR (ankle prosthe*[Title/Abstract])) OR (elbow prosthe*[Title/Abstract])) OR (finger prosthe*[Title/Abstract])) OR (joint prosthe*[Title/Abstract]) |
| #5 | #1 OR #2 OR #3 OR #4 |
| #6 | Search:("Cryotherapy"[Mesh]) OR ("Temperature"[Mesh] OR "Cold Temperature"[Mesh]) |
| #7 | ((Search ((((((((((cryotherap*[Title/Abstract]) OR (cold therap*[Title/Abstract])) OR (ice pack[Title/Abstract])) OR (Cryocompression therap*[Title/Abstract])) OR (Cold pack[Title/Abstract])) OR (Cool pack[Title/Abstract])) OR (Cold compres*[Title/Abstract])) OR (gel pack[Title/Abstract])) OR (Ice bag[Title/Abstract])) OR (Cryo[Title/Abstract])) OR (Cool*[Title/Abstract]) ) OR (thermal*[Title/Abstract])) OR (temperature*[Title/Abstract]) |
| #8 | #6 OR #7 |
| #9 | Search ("Randomized Controlled Trial" [Publication Type]) OR "Controlled Clinical Trial" [Publication Type] |
| #10 | Search ((random*[Title/Abstract]) OR trial[Title/Abstract]) OR groups[Title/Abstract] |
| #11 | #9 OR #10 |
| #12 | #5 AND #8 AND #11 |
| #13 | Filters: Publication date to 2022/09/12.  Items found: 631 |

| 2. Embase | |
| --- | --- |
| #1 | 'arthroplasty'/exp OR 'replacement'/exp OR 'hip arthroplasty'/exp OR 'hip replacement'/exp OR 'knee arthroplasty'/exp OR 'knee replacement'/exp OR 'ankle arthroplasty'/exp OR 'ankle replacement'/exp OR 'replacement arthroplasty'/exp OR 'shoulder replacement'/exp OR 'elbow replacement'/exp OR 'finger replacement'/exp OR 'elbow arthroplasty'/exp OR 'total hip replacement'/exp OR 'total knee arthroplasty'/exp OR 'shoulder arthroplasty'/exp OR 'total shoulder arthroplasty'/exp OR 'total arthroplasty'/exp OR 'finger arthroplasty'/exp |
| #2 | 'prosthesis'/exp OR 'knee prosthesis'/exp OR 'elbow prosthesis'/exp OR 'shoulder prosthesis'/exp OR 'ankle prosthesis'/exp OR 'joint prosthesis'/exp OR 'hip prosthesis'/exp OR 'finger prosthesis'/exp |
| #3 | arthroplast*:ti,ab,kw OR replac*:ti,ab,kw OR 'knee arthroplast*':ti,ab,kw OR 'hip arthroplast*':ti,ab,kw OR 'knee replac*':ti,ab,kw OR 'hip replac*':ti,ab,kw OR 'shoulder replac*':ti,ab,kw OR 'shoulder arthroplast*':ti,ab,kw OR 'elbow arthroplast*':ti,ab,kw OR 'elbow replac*':ti,ab,kw OR 'finger replac*':ti,ab,kw OR 'finger arthroplast*':ti,ab,kw OR 'ankle arthroplast*':ti,ab,kw OR 'ankle replac*':ti,ab,kw OR 'joint arthroplast*':ti,ab,kw OR 'joint replac*':ti,ab,kw OR 'replacement arthroplast*':ti,ab,kw |
| #4 | prosthe*:ti,ab,kw OR joint prosthe*:ti,ab,kw OR knee prosthe*:ti,ab,kw OR hip prosthe*:ti,ab,kw OR shoulder prosthe*:ti,ab,kw OR ankle prosthe*:ti,ab,kw OR elbow prosthe*:ti,ab,kw OR finger prosthe*:ti,ab,kw |
| #5 | #1 OR #2 OR #3 OR #4 |
| #6 | 'cryotherapy'/exp OR 'ice pack'/exp OR 'pack ice'/exp OR 'cold compress'/exp |
| #7 | 'temperature'/exp |
| #8 | cryotherap*:ti,ab,kw OR 'cold therap*':ti,ab,kw OR 'ice pack':ti,ab,kw OR 'pack ice':ti,ab,kw OR 'cryocompression therap*':ti,ab,kw OR 'cold compres*':ti,ab,kw OR 'cold pack':ti,ab,kw OR 'cool pack':ti,ab,kw OR cool*:ti,ab,kw OR 'cold compressive':ti,ab,kw OR 'gel pack':ti,ab,kw OR 'ice bag':ti,ab,kw OR cryo:ti,ab,kw OR temperature*:ti,ab,kw |
| #9 | #6 OR #7 OR #8 |
| #10 | 'randomized controlled trial'/exp OR 'controlled clinical trial'/exp |
| #11 | random*:ti,ab,kw OR trial:ti,ab,kw OR groups:ti,ab,kw |
| #12 | #10 OR #11 |
| #13 | Age:([adult]/lim OR [aged]/lim OR [middle aged]/lim OR [very elderly]/lim OR [young adult]/lim) |
| #14 | #5 AND #9 AND #12 AND #13  960 results for search #14 |

| 3. Web of Science | |
| --- | --- |
| #1 | TI=(arthroplast*) OR AB=(arthroplast*) OR TI=(replac*) OR AB=(replac*) OR TI=(knee arthroplast*) OR AB=(knee arthroplast*) OR TI=(knee replac*) OR AB=(knee replac*) OR TI=(hip arthroplast*) OR AB=(hip arthroplast*) OR TI=(hip replac*) OR AB=(hip replac*) OR TI=(shoulder arthroplast*) OR AB=(shoulder arthroplast*) OR TI=(shoulder replac*) OR AB=(shoulder replac*) OR TI=(elbow arthroplast*) OR AB=(elbow arthroplast*) OR TI=(elbow replac*) OR AB=(elbow replac*) OR TI=(finger arthroplast*) OR AB=(finger arthroplast*) OR TI=(finger replac*) OR AB=(finger replac*) OR TI=(ankle arthroplast*) OR AB=(ankle arthroplast*) OR TI=(ankle replac*) OR AB=(ankle replac*) OR TI=(prosthe*) OR AB=(prosthe*) OR TI=(joint prosthe*) OR AB=(joint prosthe*) |
| #2 | TI=(cryotherap*) OR AB=(cryotherap*) OR TI=(cold therap*) OR AB=(cold therap*) OR TI=(Ice pack) OR AB=(Ice pack) OR TI=(Cryocompression therap*) OR AB=(Cryocompression therap*) OR TI=(Cold pack) OR AB=(Cold pack) OR TI=(Cool*) OR AB=(Cool*) OR TI=(Cold compression) OR AB=(Cold compression) OR TI=(Cold compressive) OR AB=(Cold compressive) OR TI=(gel pack) OR AB=(gel pack) OR TI=(Ice bag) OR AB=(Ice bag) OR TI=(Cryo) OR AB=(Cryo) |
| #3 | TI=(Randomized Controlled Trial) OR AB=(Randomized Controlled Trial) OR TI=(Controlled Clinical Trial) OR AB=(Controlled Clinical Trial) OR TI=(random* ) OR AB=(random* ) OR TI=(trial) OR AB=(trial) OR TI=(groups) OR AB=(groups) |
| #4 | Languages:English or Chinese |
| #5 | #1 AND #2 AND #3 AND #4  1000 results for search #5 |

| 4.Cochrane Library | |
| --- | --- |
| #1 | MeSH descriptor: [Arthroplasty] explode all trees |
| #2 | MeSH descriptor: [Arthroplasty, Replacement] explode all trees |
| #3 | MeSH descriptor: [Prostheses and Implants] explode all trees |
| #4 | (joint arthroplast*):ti,ab,kw OR (joint replac*):ti,ab,kw OR (replac*):ti,ab,kw OR (arthroplast*):ti,ab,kw OR (knee arthroplast*):ti,ab,kw OR (knee replac*):ti,ab,kw OR  (hip arthroplast*):ti,ab,kw OR (hip replac*):ti,ab,kw OR (shoulder arthroplast*):ti,ab,kw OR (shoulder replac*):ti,ab,kw OR (ankle arthroplast*):ti,ab,kw OR (ankle replac*):ti,ab,kw OR (elbow arthroplast*):ti,ab,kw OR (elbow replac*):ti,ab,kw OR (finger arthroplast*):ti,ab,kw OR (finger replac*):ti,ab,kw OR (prosthe*):ti,ab,kw OR (joint prosthe*):ti,ab,kw OR (knee prosthe*):ti,ab,kw OR  (hip prosthe*):ti,ab,kw OR (shoulder prosthe*):ti,ab,kw OR (elbow prosthe*):ti,ab,kw OR (ankle prosthe*):ti,ab,kw OR (finger prosthe*):ti,ab,kw |
| #5 | #1 OR #2 OR #3 OR #4 |
| #6 | MeSH descriptor: [Cryotherapy] explode all trees |
| #7 | MeSH descriptor: [Temperature] explode all trees |
| #8 | (cold therap*):ti,ab,kw OR (cryotherap*):ti,ab,kw OR (ice pack):ti,ab,kw OR (Cryocompression therap*):ti,ab,kw OR (Cold pack):ti,ab,kw OR (Cool pack):ti,ab,kw OR (Cool*):ti,ab,kw OR (Cold compressive):ti,ab,kw OR (gel pack):ti,ab,kw OR (Ice bag):ti,ab,kw OR (Cool):ti,ab,kw OR (Cryo):ti,ab,kw OR (temperature*):ti,ab,kw OR (thermal*):ti,ab,kw |
| #9 | #6 OR #7 OR #8 |
| #10 | #5 AND #9 (Custom date range: Publication date to 31/07/2020)  538 results for search #7 |

| 5. CINAHL | |
| --- | --- |
| S1 | (MH "Arthroplasty, Replacement") OR (MH "Arthroplasty, Replacement, Knee") OR (MH "Arthroplasty, Replacement, Hip") OR (MH "Arthroplasty, Replacement, Ankle") OR (MH "Arthroplasty, Replacement, Shoulder") OR (MH "Arthroplasty, Replacement, Elbow") |
| S2 | (MH "Orthopedic Prosthesis+") OR (MH "Joint Prosthesis+") |
| S3 | TI arthroplast* OR AB arthroplast* OR TI replac* OR AB replac* OR TI knee replac* OR AB knee replac* OR TI knee arthroplast* OR AB knee arthroplast* OR TI hip arthroplast* OR AB hip arthroplast* OR TI hip replac* OR AB hip replac* OR TI shoulder arthroplast* OR AB shoulder arthroplast* OR TI shoulder replac* OR AB shoulder replac* OR TI elbow arthroplast* OR AB elbow arthroplast* OR TI elbow replac* OR AB elbow replac* OR TI finger replac* OR AB finger replac* OR TI finger arthroplast* OR AB finger arthroplast* OR TI ankle replac* OR AB ankle replac* OR TI ankle arthroplast* OR AB ankle arthroplast* OR TI knee prosthe* OR AB knee prosthe* OR TI hip prosthe* OR AB hip prosthe* OR TI shoulder prosthe* OR AB shoulder prosthe* OR TI elbow prosthe* OR AB elbow prosthe* OR TI finger prosthe* OR AB finger prosthe* OR TI ankle prosthe* OR AB ankle prosthe* OR TI prosthe* OR AB prosthe* OR TI joint prosthe* OR AB joint prosthe* |
| S4 | S1 OR S2 OR S3 |
| S5 | (MH "Cryotherapy") OR (MH "Temperature+") |
| S6 | TI cryotherap* OR AB cryotherap* OR TI cold therap* OR AB cold therap* OR TI Ice pack OR AB Ice pack OR TI Cryocompression therap* OR AB Cryocompression therap* OR TI Cold pack OR AB Cold pack OR TI Cool* OR AB Cool* OR TI Cold compression OR AB Cold compression OR TI Cold compressive OR AB Cold compressive OR TI gel pack OR AB gel pack OR TI Ice bag OR AB Ice bag OR TI Cryo OR AB Cryo OR TI temperature* OR AB temperature* OR TI thermal* OR AB thermal* |
| S7 | S5 OR S6 |
| S8 | (MH "Randomized Controlled Trials+") OR (MH "Clinical Trials+") |
| S9 | TI((random* OR trial OR groups)) OR AB ((random* OR trial OR groups)) |
| S10 | S8 OR S9 |
| S11 | S4 AND S7 AND S10  Items found: 232 |
